# EffectorFisher: association of disease phenotype with pangenomic protein-isoform profiles for improved prediction of fungal pathogenicity effectors

**DOI:** 10.1038/s41598-026-43646-x

**Published:** 2026-03-11

**Authors:** Mohitul Hossain, Naomi Gray, Pavel Misiun, Kristina Gagalova, Eiko Furuki, Kasia Clarke, Leon Lenzo, Hossein Golzar, Manisha Shankar, Huyen Phan, James Hane

**Affiliations:** 1 Centre for Crop and Disease Management (CCDM), Curtin University, Perth, Australia; 2 Department of Primary Industries and Regional Development (DPIRD), Perth, Australia; 3 Analytics for the Australian Grain Industry (AAGI), Curtin University, Perth, Australia

**Keywords:** Computational biology and bioinformatics, Genetics, Microbiology, Plant sciences

## Abstract

Plant-pathogenic fungi cause crop disease via a range of secreted effector proteins that interact with specific receptors of host plant cells. Effector identification can enable the diagnosis of disease outcomes and enable selection or breeding of disease-resistant crop cultivars. Bioinformatic methods have been developed to predict proteins with ‘effector-like’ properties, but the resulting number of candidates tends to be larger than can be feasibly validated and may contain numerous false positives. Challenges to effector discovery include the obfuscating effects of genome-wide mutations common to Fungi, such as Repeat-Induced Point (RIP) mutations. Refining effector predictions by incorporating disease phenotyping into genome-wide association studies (GWAS) have had mixed success for a handful of pathogen species. But the utility of GWAS approaches may be limited by low ‘signal-to-noise’ caused by widespread RIP-like SNP mutations across the genomes of most fungal pathogens. This study presents an alternative method for effector candidate refinement called ‘EffectorFisher’. EffectorFisher extends the output of Predector – a tool that automates and combines results of several bioinformatic tools commonly used in effector discovery – to apply pangenome-derived protein-isoform profiling to remove candidate effector protein isoforms with weak association with virulent phenotypes. This method was benchmarked using corresponding pangenome and phenotype data for two model wheat pathogens, each with multiple known effectors: the necrotroph *Parastagonospora nodorum* and the hemibiotroph *Zymoseptoria tritici*. Compared to prior methods based on effector-like protein properties, EffectorFisher improved predicted rankings of known effectors and reduced the total number of effector candidates. We present EffectorFisher (https://github.com/ccdmb/EffectorFisher-core) as a useful tool for refining effector predictions with phenotype data, which is broadly applicable to many fungal pathogen species, and is capable of predicting effectors involved in both gene-for-gene and inverse gene-for-gene effector-receptor interactions.

**Supplementary Information:**

The online version contains supplementary material available at 10.1038/s41598-026-43646-x.

## Introduction

The pursuit of crop yield and quality is significantly impacted by fungal plant-pathogens, with five major species alone causing crop diseases conservatively estimated to destroy what could otherwise feed > 7.5% of the current global population^1,2^. Fungal pathogens typically cause crop disease via “effector” molecules which interact with components of the plant host- cell to promote infection^3–5^. The identification of fungal pathogenicity effectors^4–6^, modes of action, and their cognate host-cell targets^7,8^, are key to building understanding and developing solutions to protect against crop disease^4,5,9^. Most known fungal effectors are low-molecular weight, cysteine-rich, sequence-diverse proteins^4,5^ that are distinct from pathogen-associated molecular patterns (PAMPs) which are recognised by the host immune system. PAMP recognition leads to PAMP-triggered immunity (PTI) and confers innate or basal resistance, whereas effector recognition leads to effector-triggered immunity (ETI) due to interactions between specific effectors and cognate plant-host immune receptors^7,8^, which are typically nucleotide binding-site leucine-rich repeats (NBS-LRR) proteins^10^. Cognate effector-receptor interactions can be further sub-divided into two types: “gene-for-gene” (GFG) and “inverse gene-for-gene” (IGFG). GFG interactions involve the recognition of an effector resistance (R) receptors that trigger host-cell death^7^. IGFG interactions involve the recognition of an effector by susceptibility (S) receptors^8,11^. GFG is observed for avirulence effectors (AVRs) of biotrophs and hemibiotrophs, where immune-recognition leading to host-cell death prevents further infection. Conversely, IGFG is commonly observed for necrotrophic effecotrs (NEs) - where host-cell death is an advantageous outcome for a necrotroph. Diagnostically-useful effectors will exhibit differential resistance or susceptibility between different host cultivars, indicating that cultivars vary in their receptor contents, and that optimally-resistant cultivars can be selected to match the effector profile of a local pathogen population.

Effector discovery has been accelerated by recent advancements in bioinformatics, effector prediction, and growing genomic resources for plant-pathogenic fungi^3,4,9,12^, yet significant challenges remain. The non-fungal taxonomic group Oomycetes contains microbial pathogen species that produce effector proteins with similar properties to fungal effectors^9,13^. Detection of effector motifs from the Oomcyetes has been rapid due to strong sequence conservation^14–18^, but similar representation of fungal effectors, domains and motifs within public databases^19–21^ has progressed slowly by comparison. Filamentous fungal genomes typically accumulate genome-wide mutations (such as repeat-induced point mutation (RIP)^22,23^ which are not observed in Oomycetes, and consequently their fungal effector proteins are sequence-diverse^3^. Recent studies modelling their three-dimensional structures of fungal effectors have made interesting progress, proposing new groupings based on structural-homology^24–29^. Despite this, many effectors still lack known structural homology^3,27^. Current effector prediction methods reduce the whole fungal proteome using a series of bioinformatic filters to predict a sub-set of candidate secreted effector proteins (CSEPs) that have secreted, low-molecular weight, cysteine-rich, and/or effector-like properties^4,5,9,12^. Recently, various bioinformatic tools and databases commonly used for CSEP prediction including SignalP^30^, EffectorP^30,31^, ApoplastP^32^, DeepPredEff^33^, PHIbase^34^ and InterPro^35^, have been aggregated into the Predector pipeline^36^, which predicts an overall score that can be used to rank the “effector-likelihood” of candidates. Although these approaches have assisted effector discovery, further improvements to accuracy and reduced numbers of candidates are still needed to enable rapid experimental validation. With current plant pathogen genome studies reporting hundreds of predicted CSEPs even when applying a combination of cutting-edge approaches^37^, the generation of CSEP lists that are as accurate and small as possible remains an ongoing challenge in plant pathology.

Disease phenotype data has strong potential to provide additional support for CSEP prediction, with ‘genome-wide association studies’ (GWAS) being the conventional approach^38^. GWAS for effector discovery requires: a) a population of differentially-virulent pathogen isolates, from which single-nucleotide polymorphism (SNP) variation data is generated and tested for phenotypic-association versus b) a panel of differentially-resistant host cultivars/lines with quantitative disease phenotype scores versus pathogen isolates from (a). GWAS-based effector prediction has been successfully applied to some plant-pathogenic fungi. *Parastagonospora nodorum* GWAS studies have predicted new CSEPs, highlighted previously-identified effectors SnTox3 and SnToxA^39^, and supported the discovery of SnTox5^40^. Similarly, *Zymoseptoria tritici* GWAS studies have identified the avirulence effector AvrStb6^41^, as well as predicted new CSEPs and highlighted known effectors^42–44^. A *Cercospora beticola* study also identified the CSEP locus *AvrCR4*^45^. Broader application of GWAS to non-model species may not be feasible with the current absence of high-quality pangenomic and phenotypic data for most species. Other factors that could potentially limit GWAS analysis are sample bias for clonal populations, or low diversity due to lack of sexual recombination in some lineages^46,47^. Another significant challenge for using GWAS effectively in most Fungi is its inherent reliance on SNP variation. The occurrence of repeat-induced point mutation (RIP, predominantly CT SNP mutations)^48^ in fungal genomes can present methodological challenges by increasing background noise at the SNP-level, and RIP-like mutations have been reported across a wide range of fungal lineages^22^. Recently, widespread RIP was observed across the *P. nodorum* pangenome, where an extremely low proportion of these SNPs resulted in non-synonymous amino-acid changes^23^. Protein isoform-based approaches can offer an improved signal-to-noise ratio, as there appears to be strong selection against the effects of RIP at the amino acid level^49^. Isoform variation has also been demonstrated to directly influence pathogenicity phenotype for the *P. nodorum* effector SnToxA^50^. Outside of the Fungi, recent multidisciplinary protein- association studies (“PWAS”) have also demonstrated improved sensitivity and accuracy relative to GWAS^51–54^.

In this study we describe a “PWAS” approach for CSEP-refinement - called “EffectorFisher” - which integrates conventional disease phenotype data with protein-isoform profiles derived from pangenomic data. EffectorFisher builds upon the output of the combined CSEP prediction pipeline Predector^36^, and complements it with pangenome-derived protein isoform-profiling and disease phenotype data. EffectorFisher refines CSEP predictions by removing CSEPs with weak association with disease phenotypes, and adds further context by predicting the relative virulence and cultivar-specificity of effector protein-isoforms. To demonstrate its effectiveness, we benchmarked EffectorFisher using published sets of corresponding pangenome and phenotype data, for two model species and wheat-pathogens: the necrotrophic *P. nodorum* and the hemibiotrophic *Z. tritici*. With the potential for broad application to other fungal pathogen species in mind, we also explored minimum-viable dataset requirements using randomly-reduced subsets of the *P. nodorum* dataset. EffectorFisher is available from https://github.com/ccdmb/EffectorFisher-core.

## Results

### EffectorFisher reduced CSEP numbers and improved ranking of predictions for confirmed necrotrophic and avirulence effectors

For *P. nodorum*, three different phenotype panels were tested (see Methods, Supplementary Table 1): Phenotype-A (14 isolates, 7 cultivars), Phenotype-B (154 isolates, 12 cultivars), and Phenotype-C (PhenotypeA + B: 154 isolates, 15 cultivars). For *Z. tritici*, the phenotype panel (Phenotype-D) contained 132 isolates and 12 cultivars (Supplementary Table 1). Experimentally-validated effectors of *P. nodorum* and *Z. tritici* (Table 1) were used as positive controls to empirically-define initial CSEP prediction criteria and to evaluate reduction of the CSEP dataset by EffectorFisher. CSEP datasets were generated for the purpose of demonstrating the reduction in dataset size by using EffectorFisher. *P. nodorum* CSEPs were filtered based on empirical observations of known effectors for a predicted secretion, a Predector Score ≥ 2, amino acid length ≤ 300, and cysteine residues ≥ 2, resulting in 185 CSEPs. Subsequent filtering of *P. nodorum* CSEPs for EffectorFisher p-value ≤ 0.05 improved rankings of known NEs from 1.50 to 4.08-fold, and reduced the total number of candidates by ~ 3.70-fold, ~ 1.58-fold and ~ 1.55-fold for phenotypes A, B, and C respectively (Table 1A). *Z. tritici* CSEPs were filtered based on empirical observations of known effectors for either (a) Predector Score > 2, amino acid length < 300 and predicted secretion, or; (b) homology to a known effector (of the Predector dataset or a PHIbase match with a virulence-related phenotype), resulting in 1391 CSEPs.

**Table 1:** Comparisons between outcomes of initial Predector-based CSEP predictions (effector-like properties) and refined effector candidate predictions using EffectorFisher (disease phenotype-association), for known effectors of the necrotroph *Parastagonospora nodorum* (A) and the hemibiotroph *Zymoseptoria tritici* (B).

Description	Approx. Length (aa)	#cys	Predector Score	EffectorFisher *p*-value (lowest isoform)			CSEP Rank without EffectorFisher	EffectorFisher Improved Rank (*p* ≤ 0.05)				EffectorFisher Fold-change (*p* ≤ 0.05)		
				A	B	C		A	B	C		A	B	C
(A) *Parastagonospora nodorum* (necrotroph)														
ToxA	178	2	3.76	1.30E-02	2.20E-02	1.30E-02	3	2	3	3		1.50	.	.
Tox1	117	16	3.73	8.50E-02	1.40E-02	1.40E-02	5	.	5	5		.	.	.
Tox3	230	6	2.78	3.50E-02	9.00E-03	9.00E-03	40	13	26	26		3.08	1.53	1.54
Tox5	217	6	2.37	1.40E-02	4.60E-03	4.60E-03	106	26	67	68		4.08	1.58	1.56
Tox267	265	10	2.34	3.50E-02	3.70E-02	3.50E-02	110	27	71	72		4.07	1.55	1.53
Total candidates	-	-	-	-	-	-	185	50	117	119		3.70	1.58	1.55

Description	Approx. Length (aa)	#cys	Predector Score	EffectorFisher *p*-value (lowest isoform)			CSEP Rank without EffectorFisher	EffectorFisher Improved Rank (*p* ≤ 0.0005)			EffectorFisher Fold-change (*p* ≤ 0.0005)			
				D				D			D			
(B) *Zymoseptoria tritici* (hemibiotroph)														
AvrStb6	83	12	3.9	3.60E-05			2	1			2.00			
Zt6	139	2	3.09	6.20E-05			31	18			1.72			
Zt2	167	1	2.8	4.10E-10			66	40			1.65			
Zt9	109	7	2.54	4.00E-03			107	-			-			
ZtNIP2	179	4	2.42	8.40E-02			145	-			-			
Mg1LysM	77	3	2.19	1.10E-05			221	143			1.55			
Zt10	200	4	2.13	2.80E-04			239	153			-			
Mg3LysM	234	9	2.07	3.90E-09			256	161			1.59			
Avr3D1	94	8	2.063	1.26E-09			261	166			1.57			
ZtNIP1	166	4	2.01	3.20E-08			281	178			1.58			
Zt11	87	9	1.953	9.18E-07			285	182			1.57			
Zt7	157	4	1.84	7.70E-02			290	-			-			
Zt4	150	4	1.8	6.90E-05			293	186			1.58			
Zt3	346	3	1.18	2.20E-09			311	196			1.59			
Zt15	160	1	1	2.80E-03			317	-			-			
Zt5	307	6	0.62	2.10E-04			333	210			1.59			
AvrStb9	716	8	0.24	4.12E-06			380	240			1.58			
ZtSSP2	201	10	−0.55	8.05E-02			466	-			-			
Zt89160	374	7	−0.78	7.60E-12			488	310			1.57			
*Zt80707	212	0	−1.59	6.20E-04			600	-			-			
*Zt8	267	5	−1.86	1.00E-03			648	-			-			
*Zt103264	186	2	−2.36	2.60E-03			764	-			-			
*Zt1	746	9	−3.35	6.60E-07			1324	823			1.61			
Total candidates							1391	868			1.60			

* <50% identical to known effector.

**Fig. 1:**
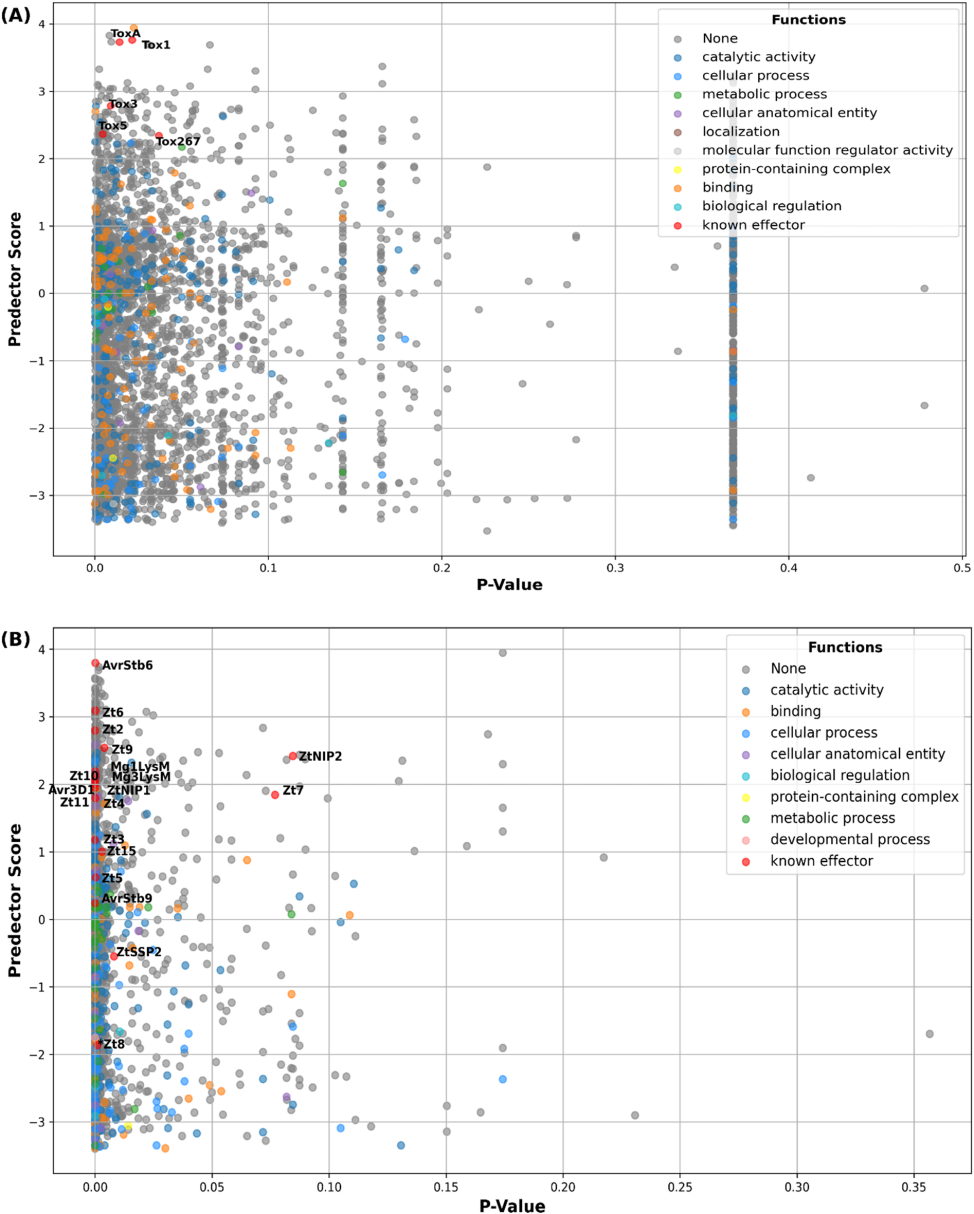
Predicted secretomes of the necrotroph *Parastagonospora nodorum* (**A**) and the hemibiotroph *Zymoseptoria tritici* (**B**), indicating the distribution of EffectorFisher (phenotype-association) p-values (x-axis) relative to Predector scores indicating effector-like properties (y-axis). Broad functional annotation categories are color-coded (see legend) with confirmed effectors indicated by a red star.

ZtNIP2 and Zt7 were excluded as outliers as they exhibited no sequence variability across the *Z. tritici* pangenome (with p-values of 8.4E-2 and 7.7E-2, Table 1B and Fig. 1B). Based on empirical observations, *Z. tritici* CSEPs were more stringently filtered for p-value ≤ 0.0005 resulting in a 1.6-fold reduction of total candidates from 1391 to 868, and a rank-improvement of known AVRs from 1.55 to 2-fold. Overall, known *P. nodorum* NEs were observed to have higher phenotype-associated p-values (4.6E-3 to 8.5E-2) and higher Predector scores indicating effector-like properties (2.34 to 3.76) relative to known AVRs of *Z. tritici* (excluding outliers, p-values: 7.6E-12 to 4E-3; Predector scores: −3.35 to 3.8) (Table 1B, Fig. 1, Supplementary Figs. 1, 2, 3).

### Prediction of cultivar-specificity at locus and protein-isoform levels

**Fig. 2:**
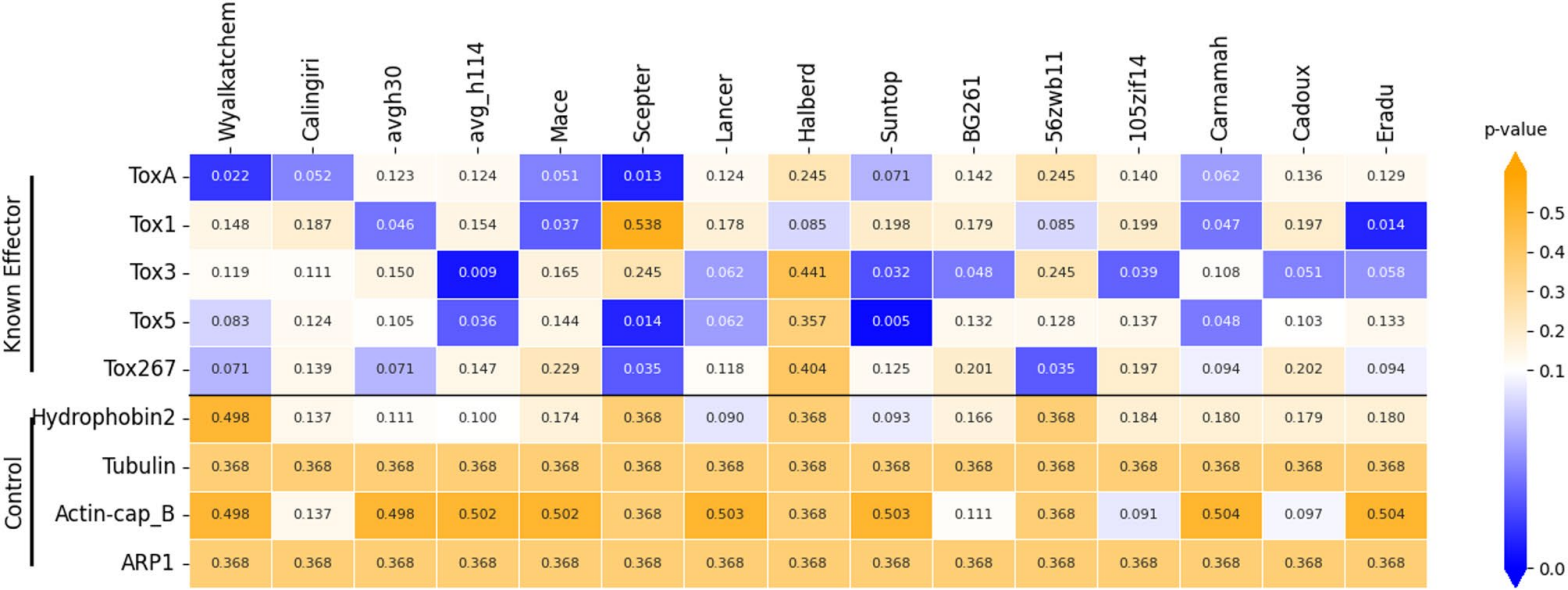
Cultivar-specific association analysis p-values of known *P. nodorum* necrotrophic effectors vs. non-effectors, summarised at the locus level, versus disease phenotyping panel “phenotype-C”.

**Fig. 3:**
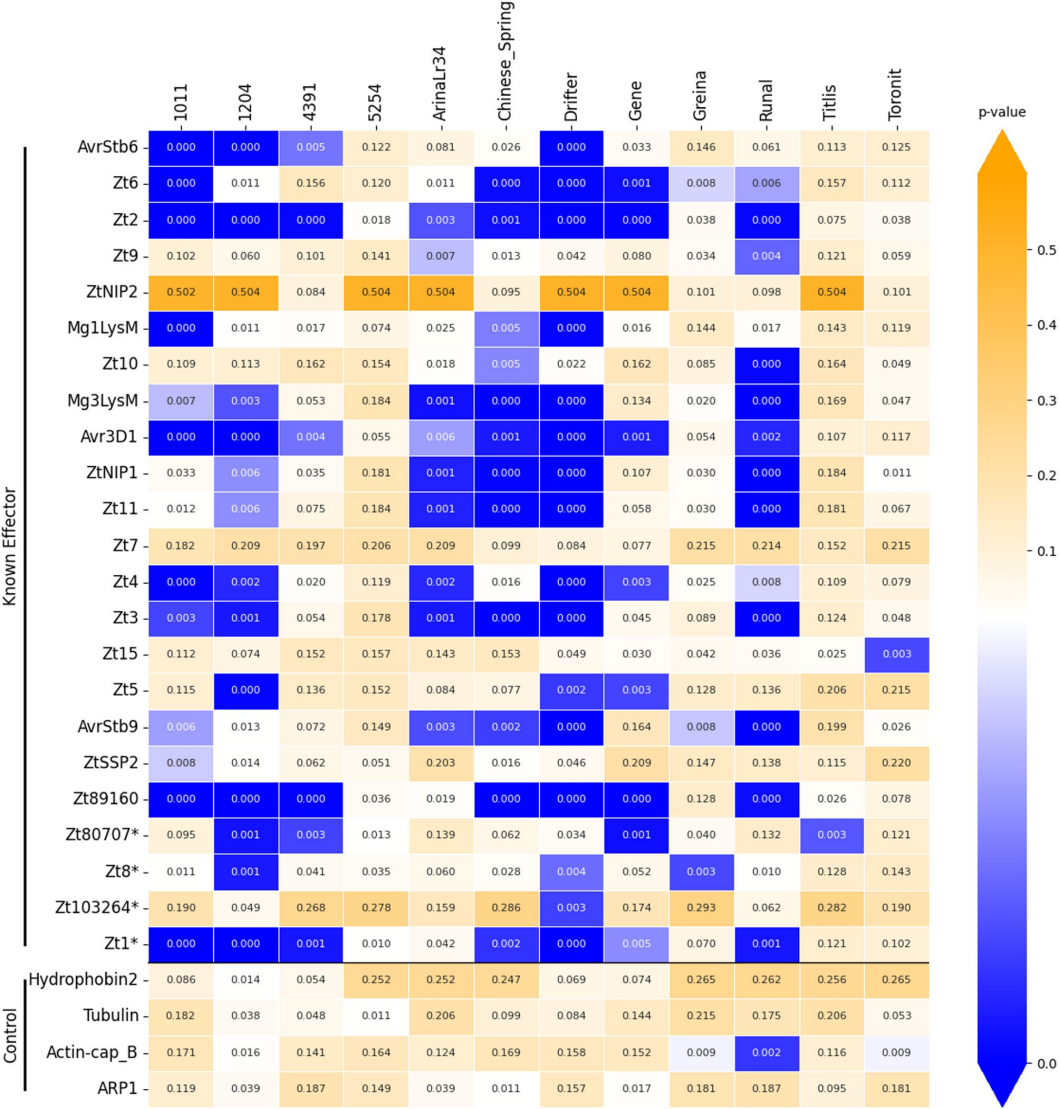
Cultivar-specific association analysis p-values of known *Z. tritici* avirulence effectors vs. non-effectors, summarised at the locus level, versus disease phenotyping panel “phenotype-D”.

Testing EffectorFisher on known NEs of the necrotroph *P. nodorum* and AVRs of the hemibiotroph *Z. tritici*, predicted a series of p-values for different combinations of effector isoforms and cultivars. For each effector-cultivar combination, the lowest p-values were interpreted as predicting higher virulence (i.e. indicating either NE susceptibility or AVR avirulence interactions) and highest p-values were interpreted as predicting low/non-virulence. The lowest p-value for each effector ortholog group was used to predict effector-cultivar interactions at the locus level, for both *P. nodorum* versus the “Phenotype-C” panel (Fig. 2) and *Z. tritici* versus the “Phenotype-D” panel (Fig. 3). Locus-level results for *P. nodorum* versus phenotype panels “Phenotype-A” and “Phenotype-B” are presented in Supplementary Figs. 4 and 5, and p-values for individual effector protein-isoforms corresponding to the above are presented in Supplementary Figs. 6, 7, 8 and 9.

Predicted association of the same effector-cultivar combinations varied depending on the cultivar panel, as observed for results across *P nodorum* phenotype datasets A-C. The first two panels (Phenotype-A and -B) were relatively distinct with few cultivars in common, whereas the third (-C) was a combination of the first two (Supplementary Fig. 10). Common cultivars allowed for limited comparison of prediction sensitivity for known NEs. Sensitivity to SnTox3 and SnTox267 was predicted (p ≤ 0.05) for cv. ‘Mace’ versus the Phenotype-A panel, but not for the Phenotype-B panel. Similarly, cv. ‘Wyalkatchem’ was predicted as sensitive to SnToxA, SnTox5 and SnTox267 (*p* ≤ 0.1) versus Phenotype-B, whereas Phenotype-A predicted sensitivity to SnTox1.

### GWAS was unable to directly identify known *P. nodorum* effectors

Variant calling and filtering resulted in 344,287 SNPs, and further filtering for linkage disequilibrium (LD) < 50% resulting in 55,498 SNPs. GWAS was conducted on SNPs with (Supplementary Fig. 11) and without LD filtering (Supplementary Fig. 12), with LD-filtering providing better results (described below). GWAS analysis was applied to *P. nodorum* versus the “Phenotype-B” panel, with both simple linear mixed models (LMMs) and multi-locus LMMs, however using simple LMMs did not produce any significant SNPs. GWAS analysis using multi-locus linear-mixed models predicted 6 ortholog groups corresponding to SNPs with significant p-values (SNOO_08097, SNOO_09331, SNOO_09364, SNOO_10769, SNOO_13986, SNOO_307520) (Supplementary Fig. 11, Supplementary Table 2). None of these ortholog groups directly corresponded to known effectors, or had functional annotations related to virulence. However identification of significant SNPs by GWAS within the proximity of a gene locus in some isolates may also indicate a potential effector. In the case of the SNP associated with locus *SNOG_13986*, which is located near the end of Chromosome 7 in the SN15 reference isolate, we observed potential linkage with the *SnTox5* locus, which is not present in SN15 but has been reported within Chr7-homologous regions of alternate isolates^40^.

### Reduced Dataset analysis indicated minimum viable data required for EffectorFisher

To test the potential for EffectorFisher to be re-applied to data from a novel pathogen species, we analysed randomly reduced subsets of the *P. nodorum* pangenome versus the Phenotype-B panel. Two scenarios were tested: (i) reduced numbers of cultivars and/or isolates, and ii) an extreme case where disease phenotype data was non-quantitative and only 1-cultivar (i.e. cultivar of isolation) was available for each isolate.

**Fig. 4:**
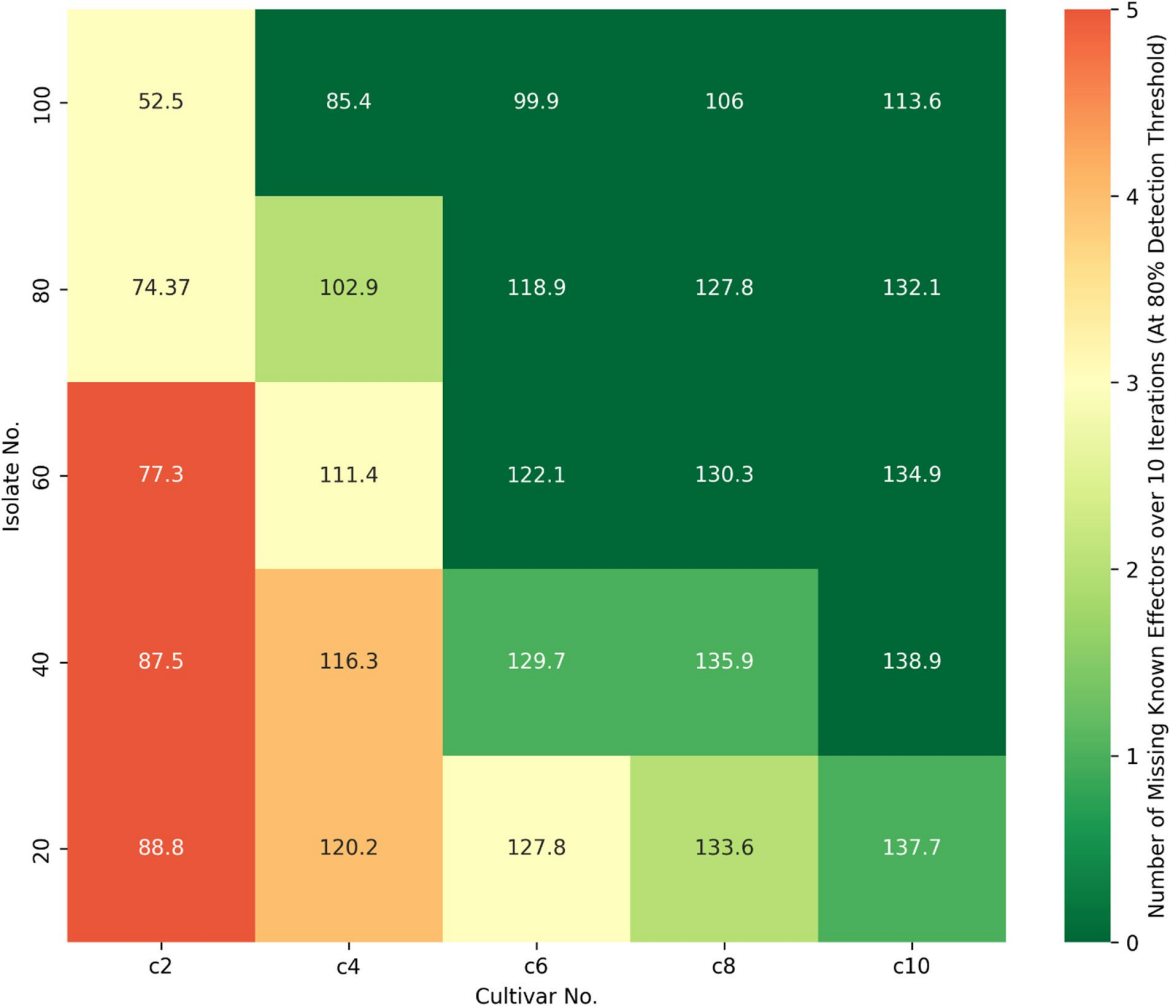
Tabular heatmap showing averaged results of 10 iterations of reduced combinations of 20–100 isolates versus 2–10 cultivars, using an EffectorFisher p-value threshold of 0.1. The heatmap color gradient from red-green (see legend) indicates the number of known effectors missing (red) or present (green) in predictions derived from the reduced data, where known effectors were considered missing if not detected in  ≥ 80% of randomly reduced iterations. The number values in each cell represent the average number of CSEPs predicted from the reduced data.

**Fig. 5:**
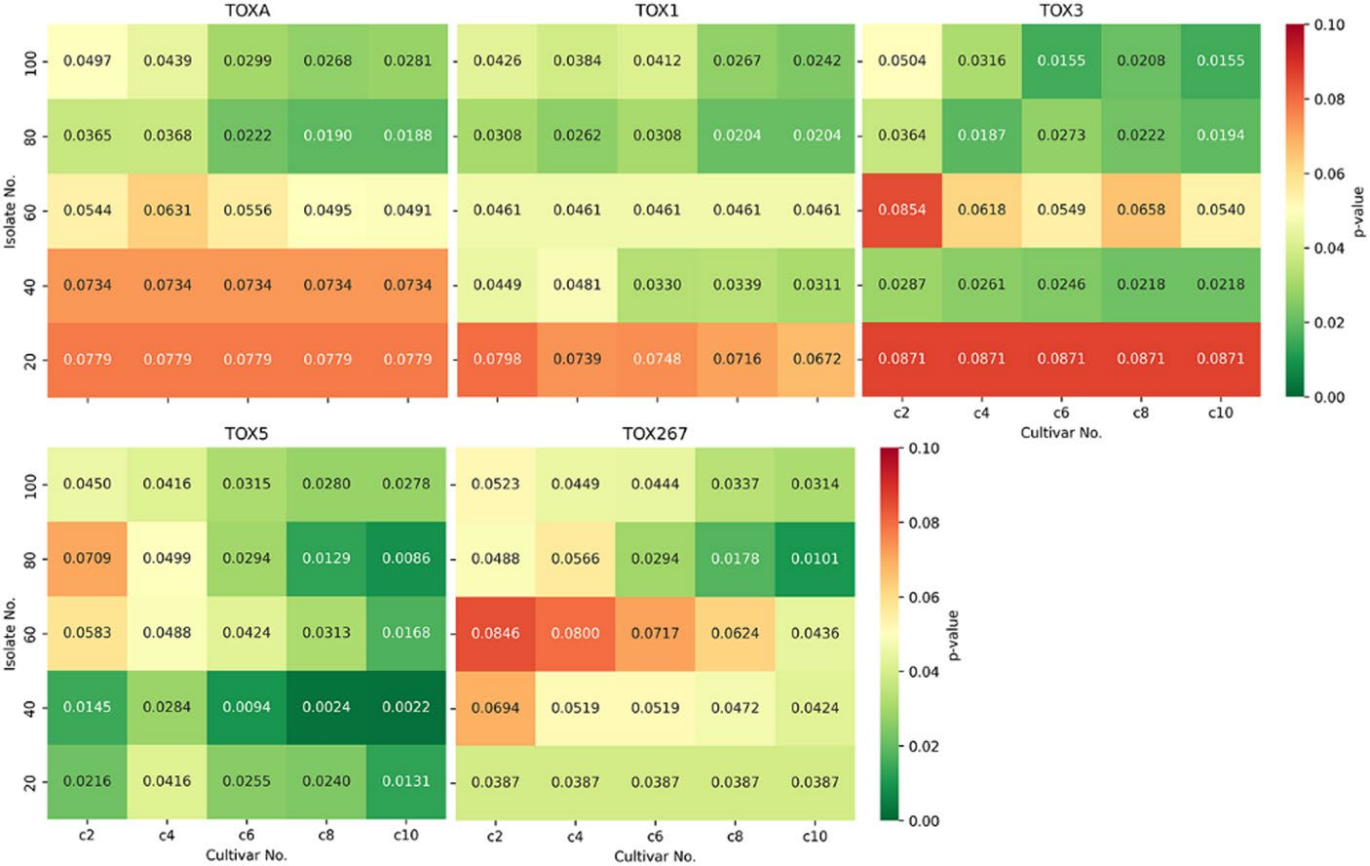
Tabular heatmaps indicating change in detection of individual known effectors with different combinations of 20–100 isolates and 2–10 cultivars. Average p-values are provided both as values and indicated by color (see legend).

Analysis of multiple combinations of 20–100 isolates and 2–10 cultivars, indicated that the number of predicted CSEPs varied depending on p-value thresholds, number of cultivars, and number of isolates (Fig. 4, Supplementary Fig. 13). As p-value decreased, the number of predicted CSEPs also decreased, but caused known effector candidates to be missed (> = 80% of the time, Supplementary Fig. 13) at low isolate (~ < 80) or cultivar numbers (~ < 6) (Fig. 4). At 2 cultivars (1S1R = 1 susceptible and 1 resistant), p-values of 0.05–0.1 missed known effectors regardless of the number of isolates. At 6 cultivars (3R3S) or more, no known effectors were missed at *p* < 0.1, but some may be missed at *p* < 0.05 (Fig. 5, Supplementary Fig. 13). At 8 cultivars (4R4S) with > 60 isolates, > 90 CSEPs were predicted with all known effectors present (Figs. 4 and 5). Results were averaged over 10 iterations, with some individual iterations performing better than average, suggesting that the selection of cultivars exhibiting distinct variations in disease phenotype relative to the effectors of interest is a critical factor for experimental design. Figure 4 used a missing-data threshold of 80%, but similar trends were observed with thresholds from 30 to 100% (Fig. 5, Supplementary Fig. 13).

Randomly sampled subsets of 12 cultivars and 9–13 isolates from the “Phenotype-B” panel were used to test an extreme case, where only a single “cultivar-of-isolation” is recorded for a set of virulent isolates. For each isolate, a single cultivar disease phenotype score greater than the median was randomly selected to represent “high” virulence for that isolate, and values for all other cultivars were set to “low” virulence. Non-quantitative predictions were comparable to predictions from the full Phenotype-B panel (Supplementary Table 2), resulting in up to 4.6-fold improvement of known effector rank (cf. 4.9-fold) and 3.38-fold reduction in total CSEPs (versus 3.7-fold). It should be noted a higher p-value threshold was set at 0.1 to accommodate the limitations of the non-quantitative dataset.

## Discussion

EffectorFisher is a new method, extending upon the combined effector prediction pipeline Predector, using pangenome-derived protein-isoform profiles of multiple pathogen isolates, to test for their association with quantitative disease phenotype data versus a panel of host cultivars. Its intended purpose is the refinement of conventionally-predicted CSEPs with effector-like properties^4,5,9^, removing effector-like CSEPs with weak association with disease phenotype, thereby generating smaller, more accurate sets of CSEPs likely to be strongly-associated with disease phenotype. EffectorFisher also tests association separately for all phenotyped cultivars, and in doing so can generate p-values that may indicate specificity versus one or more cultivars. To demonstrate its utility, we performed benchmarking versus two model wheat pathogens - *Parastagonospora nodorum* (a necrotroph) and *Zymoseptoria tritici* (a hemibiotroph). EffectorFisher was able to predict all known effectors of *P. nodorum* and several from *Z. tritici* (details below), improving their relative effector-likelihood ranks based on Predector^4^ scores and reducing the total number of CSEPs. Moderate improvements were observed even using limited non-quantitative phenotype data, i.e. cultivar of isolation (Supplementary Table 3). Analysis of functional annotations supported the refinement of both sets of CSEPs, removing candidates with predicted functions commonly associated with house-keeping and/or metabolism (Fig. 1, Supplementary Figs. 1, 2, 3).

Important differences were observed between the predictions of necrotrophic effectors (NEs, of *P. nodorum*) and avirulence effectors (AVRs, of the hemibiotroph *Z. tritici*). Known NEs were predicted to have moderate p-values and higher Predector scores, whereas known AVRs had very low p-values with a far broader range of Predector scores. EffectorFisher was also able to reduce the predicted CSEP set size for *P. nodorum* NEs to a greater extent than for *Z. tritici* AVRs (Table 1). These different prediction outcomes may reflect the biological differences between IGFG interactions between NEs and susceptibility (S) receptors, and the GFG interactions between AVRs and resistance (R) receptors. Repeat-induced point mutation (RIP) is the strongest driver of genome-wide mutations in both species^22,23,55^, which can introduce stop mutations into protein-coding sequences^48^. Rapid loss-of-function mutations due to RIP plays a significant and beneficial role in promoting the emergence of hemibiotroph isolates that become virulent against formerly-resistant cultivars^56^. RIP mutation is also a strong driver of genome adaptation in necrotrophs, however changes to amino acid sequences are strongly selected against^57^. Furthermore, NE loss-of-function does not confer the same benefits to a necrotroph as a functional effector protein is required to interact with its cognate host S receptor in order to cause disease symptoms. The different biological consequences of amino acid mutations for NEs and AVRs may explain the moderate phenotype-association p-values observed for the protein-isoforms of the necrotroph and strong p-values of those of the hemibiotroph. Based on these findings and our empirical observations of known effectors in each species, we suggest AVR-discovery approaches should apply stricter filtering on p-values (e.g. ≤0.0005) whereas NE-discovery approaches should apply standard thresholds (≤ 0.05).

For the model necrotroph *P. nodorum*, EffectorFisher reduced the number of CSEPs from 185 to 51–119 candidates (depending on the phenotype panel used). This CSEP set contained all known NEs – SnToxA^50,58,59^, SnTox1^60^, SnTox3^61^, SnTox267^62^ and SnTox5^40^ – each with specific virulence versus cultivars that possess corresponding S-genes (Supplementary Table 4, Supplementary Table 5). Functional analysis using InterProScan indicated only one (of 119) CSEP had a functional annotation (related to binding) and the majority of ortholog groups with functional-annotations had been excluded. This suggests EffectorFisher filtered out non-pathogenic false positives, including those with housekeeping and miscellaneous functions, thereby enriching for CSEPs with cultivar-specificity. This CSEP set was also compared to previously published gene expression data^63^[NCBI GEO: GSE150493] (*in planta* versus *in vitro* RNAseq of the SN15 reference isolate^57^, which highlighted those which were upregulated during infection, and providing additional supporting evidence for prioritising CSEP candidates. Of 119 CSEPs, 20 were upregulated *in planta* (Supplementary Table 2), and non-upregulated CSEPs included known effectors SnTox5 (absent in SN15) and SnTox3. More broadly, effector association profiles were observed to be similar among cultivars with common breeding lineages (Supplementary Fig. 10)^64^. Recently bred cultivars/lines also exhibited higher p-values compared to their parent cultivars, possibly suggesting a relative increase in resistance over time and with successive breeding events. There was no evidence to support using EffectorFisher p-values as a direct indicator of the relative virulence activities of different isoforms however. Prior assays of the relative activities of SnToxA isoforms used one cultivar common to this study (cv. BG261)^50^, which used a standardised nomenclature for SnToxA (DNA-)haplotypes and (protein-)isoforms^65^. In this study, SnToxA isoforms 1–4 were automatically-numbered based on descending order of frequency observed across the pangenome. SnToxA_1, SnToxA_2 and SnToxA_4 and corresponded to ToxA3 (H2), ToxA10 (H9) and ToxA5 (H4) of the standardised nomenclature, with ToxA_3 roughly corresponding to ToxA_15 (H15) (Supplementary Table 4). BG261 was used as a ‘SnToxA-differential’ line, as it contains a *Tsn1* S-gene that confers varying degrees of susceptibility to most SnToxA isoforms^50^. Higher activity (vs. cv. BG261) was reported for H4, moderate for H9 and lower for H15. EffectorFisher did not predict significant association with any isoform versus cv. BG261, with lower p-values (0.129 and 0.143) for ToxA_1 and ToxA_2, and slightly higher p-values (0.368 and 0.504) for ToxA_3 and ToxA_4. We speculate that it was not possible for this effector-cultivar combination to exhibit a clear pattern of phenotypic variation that could be detected by EffectorFisher, because BG271 is susceptible to all 4 SnToxA isoforms observed in this dataset. Nevertheless, the broad pangenomic survey approach used by EffectorFisher predicted a refined CSEP set containing all known NEs with cultivar-specificity, which may be missed in analyses of data derived from a limited number of isolates (e.g. transcriptomics).

For the model hemibiotroph *Z. tritici*, EffectorFisher prediction (*p* ≤ 0.0005) reduced the number of CSEPS from 1391 to 868 CSEP candidates (Supplementary Table 6). The number of *Z. tritici* CSEPs in our example dataset was relatively high due to empirically-derived prediction criteria of several known effectors (e.g. Zt8 and Zt1, Table 1) necessitating high Predector score, long length and no cysteine thresholds, however for illustrative purposes it serves to demonstrate that EffectorFisher was able to reduce the number of CSEP candidates. EffectorFisher predictions excluded known effectors Zt9, ZtNIP2, Zt7, Zt15, ZtSSP2, Zt80707, Zt8 and Zt103264, that were predicted to have weak associations with cultivar-specific disease phenotypes (*p* > 0.0005, Table 1; Fig. 3). ZtNIP2 and Zt7 were also the only known effectors with only a single protein-isoform observed across the whole pangenome, indicating strong sequence conservation and decreasing their likelihood of cultivar-specificity. The known effectors predicted by EffectorFisher included (in rank order) AvrStb6, Zt6, Zt2, Mg1LysM, Zt10, Mg3LysM, Avr3D1, ZtNIP1, Zt11, Zt4, Zt3, Zt5, AvrStb9, Zt89160, and Zt1, which were improved in rank with EffectorFisher by 1.55 to 2-fold (Table 1; Fig. 3). The top-ranked (#1) candidate was ZtAvrStb6^41^, a well-studied effector which has a critical avirulence role as host-recognition triggers stomatal closure which then impedes hyphal penetration^66^. Its corresponding host receptor Stb6 is common among commercial wheat cultivars (cv.) globally^67^, and the recent breakdown of *Stb6*-mediated resistance is a major concern. The cultivar-specificity predictions presented in Fig. 3 corroborate findings of the original study which discovered AvrStb6 via GWAS^41^, which reported differential virulence between cultivars ‘Chinese Spring’ and ‘Drifter’, however these are the only cultivars in common with the dataset used in this study, which was sourced from a later GWAS study^68^. AvrStb9, Avr3D1 and ZtSSP2 have been reported to interact with wheat host receptors Stb9^69^, Stb7/Stb12^70^ and wheat E3-ubiquitin ligase^71^ respectively. ZtNIP1 and ZtNIP2, an Ecp2 homolog and lipid-binding protein respectively, both cause necrosis in cv. Nuage, Bermude and FD3 and mild symptoms in cv. Kulm and Grandin^72^. Known effectors with the “Zt” prefix were previously screened for “non-host” virulence in *Nicotiana benthamiana*^73,74^, and for most their cultivar-specific activities on a wheat host are yet to be confirmed, however this study predicted differential p-values that may suggest resistant and susceptible cultivars (Fig. 3). The second highest-ranked known effector Zt6 is a secreted ribotoxin, with cytotoxic activity reported in wheat cultivar ‘Riband’^75^. Zt7 is also notable in having similar necrotic activity with and without its secretion signal peptide motif^73^, while Zt11 is also notable in that its pathogenicity versus wheat has been confirmed for a single cultivar (cv. Longbow)^76^. Some known effectors such as Mg1Lysm and Mg3Lysm are extracellular chitin-binding proteins which help the pathogen to evade host defences (i.e. chitinases)^77^ but do not directly interact with host-cell components, yet still exhibited cultivar-specific p-values (Fig. 3). For *Z. tritici*, to ensure the inclusion of its known effectors the CSEP dataset resulting from empirical observations was relatively large. However in practice, several targeted strategies can be practically applied to further reduce CSEP dataset size and prioritise candidates for experimental validation, including: (a) inclusion of complementary transcriptomic^78^ and/or proteomic^79^ data; (b) focus on CSEPs which through complementary comparative genomics analysis exhibit novel conservation patterns, such as lateral gene transfer^59^, (c) focusing on CSEPs with homology one or more known effectors^37,80^; (d) focus on CSEPs with higher ranked Predector scores^36^, and; (e) focus on CSEPs with predicted p-values suggesting resistance and/or susceptibility to one or more cultivars of interest (Figs. 2 and 3).

To estimate how the EffectorFisher method could be re-applied to a novel pathogen species, a series of randomly reduced datasets were analysed to determine the minimum viable number of phenotyped pathogen isolates and cultivars required. Analysis of reduced phenotype datasets suggested that upwards of 6 cultivars and 80 isolates may give useful results, with higher numbers affording greater reliability. We also observed variable outcomes between different cultivar phenotype panels. For example, the larger panel Phenotype-B predicted more effector candidates compared to the smaller panel Phenotype-A, which notably missed SnToxA (Table 1, Supplementary Figs. 4, 5). We speculate in this case that the additional cultivars added more effector-specific phenotype variation that allowed for the prediction of more significant CSEPs. However the ranking of certain known effectors was improved for the smaller panel, which may have been enriched for cultivars with differential phenotypes versus those effectors (Table 1). In testing randomised reduction of phenotype panel data to test reduced dataset sizes (Fig. 4), some iterations predicted known effectors better than average. This may inadvertently highlight the importance of optimising cultivar and isolate selection for differential effector responses. The aggregation of different phenotype panels into a larger dataset (as in “Phenotype-C”) may enable broader CSEP prediction overall, but performing multiple analyses separately may lead to more accurate and targeted results for specific sets of effectors.

Conventional GWAS approaches have been successfully applied to a handful of plant pathogen species including *P. nodorum*^39,40^, *Z. tritici*^41,68^ and *C. beticola*^45^, however the EffectorFisher method offers a few advantages. Protein-isoform profiling can provide direct interpretability and relevance to effector protein function^50^, whereas SNP-based approaches require multiple steps for filtering (e.g. non-protein-coding and synonymous SNPs) and conversion (from SNP to locus/haplotype to protein-isoform) to arrive at final CSEP predictions, and do not intrinsically report predicted cultivar-specificities. Compared to methods relying on reference-based variant calling, pangenome survey approaches also enable detection of CSEPs that are absent in the reference isolate^23^. Gene loci encoding effector proteins are also typically located near repeat-rich regions of fungal genomes that are subject to widespread RIP^22,81^ and/or structural mutations^81,82^. The negative consequences for GWAS is an elevated signal: noise ratio at the SNP level^23^ and potential breakdown of linkage disequilibrium (relied upon to associate imperfect GWAS markers with CSEP-encoding loci). While these impediments to GWAS may be accounted with careful experimental design (e.g. strong phenotype diversity, controlled genetic background and large sample size), our reduced dataset tests suggest that EffectorFisher may still be able to perform reasonably well with minimal and/or poorer quality data. Recent multi-disciplinary proteome-wide association studies (“PWAS”) outside of the Fungi have corroborated similar improvements over GWAS in sensitivity, accuracy and versatility^51–54^. Moving forward, if “pangenomic-PWAS” approaches such as EffectorFisher are to become widely-used, future challenges for the field of plant pathology will include the coordination of mirrored disease phenotype and pangenome datasets for pathogen isolate collections.

## Methods

### Pangenomic data of model necrotroph and hemibiotroph wheat pathogens

Published pangenome data (Supplementary Data 1) from two model wheat pathogen species were used in this study: (a) 14 isolates of the necrotroph *Parastagonospora nodorum* across multiple regions (Western Australia, Iran, Europe and the United States)^23,39,57,83,84^[NCBI BioProject: PRJNA686477, PRJNA398070, PRJNA476481, PRJNA170816, PRJNA170815], with reference isolates SN15^57^ [NCBI BioProject: PRJNA686477], LDN03-Sn4 (Sn4), Sn2000, and Sn79-1087^39^ [NCBI BioProject: PRJNA398070] and; (b) 132 isolates of the hemibiotroph *Zymoseptoria tritici* collected across multiple regions (Australia, Israel, Switzerland, United States)^85^[NCBI BioProject: PRJNA890236, PRJNA327615], with reference isolate IPO323^55^ [NCBI BioProject: PRJNA19047]. Additional isolates of *P. nodorum* from Western Australia (WA) were sequenced using 125 bp paired-end (PE) reads with a 600 bp insert size (Illumina HiSeq2500, TruSeq PCR-free) (Australian Genome Research Facility, Melbourne, Australia) [NCBI BioProject: PRJNA1130627], bringing the total number to 154. Whole-genomes were *de novo* assembled for all non-reference isolates with SPAdes v3.12.0^86^ (--only-assembler, --cov-cutoff auto) and MitoZ v3.6^87^ (all, --genetic_code 4, --clade Chordata, --thread_number 8, --assembler spades, --requiring_taxa Chordata, with --insert_size and --fastq_read_length adjusted according to the input data). Genome assemblies generated with SPAdes were filtered to remove sequences matching MitoZ mtDNA contigs using BLASTn^88^(≥ 95% mtDNA coverage). Filtered SPAdes and MitoZ sequences were combined into a final assembly and annotated as “nuclear” or “mtDNA” respectively. Quast v5.0.2^89^ and BUSCO v5.2.2 (database: ascomycota_odb10)^90^ were used to assess the completeness of assemblies (Supplementary Table 7).

### Gene annotation and protein-isoform profiling across two pangenomes

A set of representative sequences for potential ortholog groups were sourced from recent pangenome studies for both *P. nodorum*^23^(Supplementary Data 2) and *Z. tritici*^85^(Supplementary Data 3). These ortholog group representatives were used as input to MetaEuk v0.021^91^, which was used to annotate genome assemblies of all isolates. Pan-gene ortholog groups with zero matches after MetaEuk screening were removed from further analysis^23^, designated as the final gene annotation datasets for each isolate, and used to summarise ortholog group presence/absence (Supplementary Data 4, Supplementary Data 5). All variations of protein isoforms (unique amino acid sequences, translated from gene annotations predicted by MetaEuk) were counted across isolates for each ortholog group. Unless specified otherwise, protein-isoforms with counts < 5 across the whole pangenome were excluded from statistical tests (below).

### Prediction of Candidate Secreted Effector Proteins (CSEPs)

To predict effector-like properties, Predector^36^(v1.2.7) was first applied to the two panels of ortholog group representatives for both *P. nodorum* and *Z. tritici*. This generated ranked lists of effector candidates with summaries of effector-like properties, homology to conserved functional domains and homology to known effectors (Supplementary Table 5, Supplementary Table 6). CSEP prediction was performed separately for the two species based on empirically observed features of their respective known effectors (Table 1). *P. nodorum* ortholog groups^23^ were filtered for the following criteria: Predector score ≥ 2, cysteines ≥ 2, length ≤ 300 aa, predicted secretion (Supplementary Data 6, Supplementary Data 7, Supplementary Data 8). *Z. tritici* CSEP ortholog groups were filtered for either (Predector score ≥ 2, length ≤ 300 aa and predicted secretion) or homology to a known effector (to the known effector dataset of Predector or a PHIbase^34^ match with a virulence-related phenotype) (Supplementary Data 9). Protein isoform profiles were applied to Fisher’s exact test as in Table 2. For benchmarking, known effectors of both *P. nodorum* and *Z. tritici* (Table 1; Figs. 2 and 3), were used as positive controls, and homologs of Hydrophobin 2 MHP1, Tubulin, Actin-cap B, and ARP1 proteins were used as negative controls (Figs. 2 and 3). EffectorFisher p-value thresholds were empirically-determined to be *p* < 0.05 for *P. nodorum* (Figs. 1 and 2) and *p* < 0.00005 for *Z. tritici* (Figs. 1 and 3).

**Table 2:** Summary of disease phenotyping corresponding to *P. nodorum* and *Z. tritici* pangenome data used in this study.

Dataset Name	Pathogen Species	# Isolates	# Cultivars	Cultivar Names	Score Range	Citation
Phenotype-A	*Parastagonospora nodorum*	14	7	Cadoux, Calingiri, Carnamah, Eradu, Halberd, Mace, Wyalkatchem	0–10	^75^
Phenotype-B	*Parastagonospora nodorum*	154	12	Wyalkatchem, BG261, Suntop, 56zwb11, Lancer, H114, Mace, Scepter, Calingiri, Halberd, H30, 105zif14	0–10	This study
Phenotype-C (A + B)	*Parastagonospora nodorum*	154	15	Wyalkatchem, BG261, Suntop, 56zwb11, Lancer, H114, Mace, Scepter, Calingiri, Halberd, H30, 105zif14, Cadoux, Carnamah, Eradu	0–10	This study, ^75^
Phenotype-D	*Zymoseptoria tritici*	145	12	ArinaLr34, Chinese_Spring, Drifter, Gene, Greina, Runal, Titlis, Toronit, 1011, 1204, 4391, 5254	0–100 (converted to 0–10)	^76^

### Testing for association of protein isoforms versus disease phenotype

Two panels of disease phenotype data (Table 3) were sourced from prior studies for *P. nodorum* (designated as dataset ‘Phenotype-A’)^92^ (Supplementary Data 6) and *Z. tritici* (‘Phenotype-D’)^68^ (https://datadryad.org/dataset/doi:10.5061/dryad.j3tx95x9m) (Supplementary Data 9). Additional phenotype data for *P. nodorum* was generated (‘Phenotype B’, Supplementary Data 7), and the combination of published and new *P. nodorum* phenotype data was designated ‘Phenotype-C’ (Supplementary Data 8). Phenotype-A included 14 pathogen isolates isolates (all with sequenced genomes) versus 7 wheat cultivars (Cadoux, Calingiri, Carnamah, Eradu, Halberd, Mace, Wyalkatchem). Phenotype-B included 154 isolates versus 12 wheat cultivars (Wyalkatchem, BG261, Suntop, 56zwb11, Lancer, H114, Mace, Scepter, Calingiri, Halberd, H30, 105zif14). Of the 154 *P. nodorum* isolates with genome sequencing data: all isolates were phenotyped versus cv.’s BG261 and Wyalkatchem; 149/154 vs. cv.’s Calingiri, Halberd, and Lancer; 140/154 vs. cv.’s Mace, Sceptre and 56zbw11; and 125/154 vs. cv.’s 105zif14, avgh30 and H114. The pedigree of cultivars/lines included in Phenotype-A-C (Supplementary Data 10) was visualised using Helium v1.19.09.03^93^. Phenotype-D (Table 3) included 132 *Z. tritici* isolates isolates (all with sequenced genomes) versus 12 genetically different wheat cultivars (five landraces: Chinese Spring, 1011, 1204, 4391, and 5254; six commercial varieties: Drifter, Gene, Greina, Runal, Titlis, Toronit; and a back-cross line ArinaLr34). Disease scores were ‘semi-quantitative’, indicating an incremental progression of disease symptoms with scores ranging from 1 to 10 (Supplementary Table 1) (Phenotype-D disease scores, originally measured on a 0–100 scale, were linearly normalised to a 0–10 scale for consistency with *P. nodorum* phenotype scores). To sort isolates into binary categories for Fisher’s exact test, the median of disease scores for each panel was used as a threshold to sort isolates into ‘high-‘ and ‘low-virulence’ groups High/low virulence categories (relative to each cultivar) were compared to the protein-isoform presence/absence across isolates, using Fisher’s exact test (equation below, with multiple tests considered for each combination of cultivar and ortholog group. Summaries at the ortholog group level used the lowest p-value obtained from all protein-isoform and cultivar combinations.

$$\mathrm{p-value}=\exp \left( \log \left({a+b}\atop {a} \right)+\log \left({c+d}\atop {c} \right)-\log \left({n}\atop {a+c} \right)\right)$$

**Table 3:** Hypergeometric tests used to predict association between isoform profiles (present/absent) and disease phenotype (high/low virulence).

For each cultivar (x)	CSEP protein isoform present	CSEP protein isoform absent	Total
High Virulence	a) number of isolates with high-virulence vs. cultivar(x) with protein isoform	c) number of isolates with high-virulence vs. cultivar(x) without protein isoform	a + c
Low Virulence	b) number of isolates with low-virulence vs. cultivar(x) with protein isoform	d) number of isolates with low-virulence vs. cultivar(x) without protein isoform	b + d
Total	a + b	c + d	n

### Refining effector predictions with phenotype associations

Phenotype-based association tests were used to refine prior predictions of CSEP ortholog groups with effector-like properties. *P. nodorum* CSEP ortholog groups were filtered for ≤ 300 amino acids, ≥ 2 cysteines, Predector score ≥ 2 and (phenotype-association) p-value ≤ 0.05. *Z. tritici* ortholog groups were filtered for (a) Predector Score > 2, amino acid length >300 aa and predicted secretion, or b) homology to a known effector (of the Predector dataset or a PHIbase match with a virulence-related phenotype, with more stringent p-values at p ≤ 0.0005. The following metrics were used as indicators of the overall improvement to CSEP prediction: a) number of CSEP groups, and b) the ratio of “refined”:”initial” CSEP ranks (on descending Predector score) for known effectors of both *P. nodorum* and *Z. tritici*. To provide a functional context to the sets of genes predicted by both methods, the CSEP ortholog group representative sequences were assigned functional annotations with InterProScan v5.63–95^94^ (via Predector v1.2.7^4^), which were supplemented with hierarchical GO term information using GOATools v1.4.5 with the “go-basic.obo” ontology file (accessed July 2024)^95^. Functional annotations were used to summarise categories that were used to compare functional differences between the initial and refined CSEP datasets. Published gene expression data^63^ versus *P. nodorum* reference isolate SN15^57^, was compared to *P. nodorum* CSEP groups that were up-regulated during infection (Supplementary Table 2).

### Estimation of minimum viable dataset size

The EffectorFisher approach required two corresponding types of datasets for the same series of pathogenic isolates: (a) whole-genome sequencing, and (b) phenotyping versus host cultivars. EffectorFisher analyses presented in this study used datasets ranging from 14 to 155 isolates and 7–15 cultivars for the model species *P. nodorum* and *Z. tritici*, with few other plant-pathogen species currently as well represented. To test the minimum viable quantities for both data types, several reduced sub-sets derived from *P. nodorum* dataset-B were generated, with incrementally-reduced amounts of isolates (20, 40, 60, 80, 100) and cultivars (2(1 resistant/1 susceptible), 4(2R/2S), 6(3R/3S), 8 (4R/4S), 10(5R/5S)). Cultivars were stratified by median disease severity across all isolates and classified into low- or high-severity groups relative to the overall median; equal numbers of cultivars were then randomly subsampled from each group using fixed random seeds to ensure reproducibility. Within each selected cultivar, isolates were further stratified by cultivar-specific median severity and randomly subsampled in equal numbers from each stratum (e.g. 40 low- and 40 high-severity isolates per cultivar in the case of reduction to 80 isolates). An extreme example of non-quantitative phenotyping was also tested, where only the ‘cultivar-of-isolation’ was recorded for each isolate, which was generated by selecting one high disease-scoring cultivar at random for each pathogen isolate.

### Comparisons with Genome-Wide Association Studies (GWAS)

To demonstrate the utility of EffectorFisher predictions, comparisons to effector-focussed GWAS analyses (as per prior studies^42,44^ was performed for both *P. nodorum* and *Z. tritici*. Comparable GWAS data for *Z. tritici* was obtained from a prior study^44^. Prior GWAS studies for *P. nodorum* were also available, however for comparability to this study, new analysis needed to be performed. Raw genome sequence reads of the 154 *P. nodorum* isolates from Phenotype-B were aligned using BWA-MEM v0.7.17^96^ to the *P. nodorum* SN15 reference genome assembly^57^, and variant calling was performed with GATK v4.2.6.1^97^ (GATK HaplotypeCaller, GVCF mode; GenotypeGVCFs; VariantFiltration (SNPs): QD < 2, QUAL < 30, SOR > 3, FS > 60, MQ < 40, MQRankSum<−12.5, ReadPosRankSum<−8. A set of 868050 bi-allelic SNPs was further filtered using BCFtools v1.15^98^ for minor allele frequency (MAF) > 10% and < 5% missing data, and further filtered for linkage disequilibrium. Linkage disequilibrium (LD) pruning was performed using bcftools +prune with a pairwise r^2^ threshold of 0.5 (-m 0.5) and a window size of 5 kb (-w 5000 bp). Within each window, SNPs with r^2^ > 0.5 were iteratively pruned until all remaining pairwise r^2^ ≤ 0.5. GWAS analysis was performed using SNPs with and without LD filtering. To capture cultivar-specific genetic effects, GWAS was conducted independently for each wheat cultivar, and results were visualized using cultivar-specific Manhattan plots. For comparison to EffectorFisher results from this study, GWAS was applied to the filtered *P. nodorum* variants using both single and multi-locus linear mixed models implemented in GAPIT v3^99^ using the Efficient Mixed Model Association (EMMA) algorithm. A VanRaden-derived kinship matrix was used as a random effect (PCA “true”), to determine the optimum number of principal components (PCA.total = 3) to account for population structure and relatedness to be included in the model. Model fit was assessed via Q-Q plots of observed vs. expected -log10(p-values). Statistical significance was determined using Bonferroni correction (α = 0.05) to control for multiple testing.

## Supplementary Information

Below is the link to the electronic supplementary material.

Supplementary Material 1

Supplementary Material 2

Supplementary Material 3

Supplementary Material 4

Supplementary Material 5

Supplementary Material 6

Supplementary Material 7

Supplementary Material 8

Supplementary Material 9

Supplementary Material 10

Supplementary Material 11

Supplementary Material 12

Supplementary Material 13

Supplementary Material 14

Supplementary Material 15

## Data Availability

EffectorFisher code is available from [https://github.com/ccdmb/EffectorFisher-core](https:/github.com/ccdmb/EffectorFisher-core). Genome sequence data for *Parastagonospora nodorum* was obtained for: (a) reference isolates SN15 [NCBI BioProject: PRJNA686477], LDN03-Sn4 (Sn4), Sn2000, and Sn79-1087 39 [NCBI BioProject: PRJNA398070], (b) 14 isolates from multiple regions (Western Australia, Iran, Europe and the United States) [NCBI BioProject: PRJNA686477, PRJNA398070, PRJNA476481, PRJNA170816, PRJNA170815], and (c) 136 isolates from Western Australia [NCBI BioProject: PRJNA1130627]. Genome sequence data for *Zymoseptoria tritici *was obtained for (a) 132 isolates collected across multiple regions (Australia, Israel, Switzerland, United States) [NCBI BioProject: PRJNA890236, PRJNA327615], and (b) reference isolate IPO323 [NCBI BioProject: PRJNA19047]. Disease phenotype datasets used in this study were sourced from (a) a prior study of *P. nodorum* ([https://doi.org:10.3389/fpls.2019.01785] (https:/doi.org:10.3389/fpls.2019.01785)) (b) a prior study of *Z. tritici* by ([https://doi.org: https://doi.org/10.1111/eva.13117] (https:/doi.org: https:/doi.org/10.1111/eva.13117)) (available from: [https://datadryad.org/dataset/doi:10.5061/dryad.j3tx95 × 9 m] (https:/datadryad.org/dataset/doi:10.5061/dryad.j3tx95 × 9 m)).
