# Supplementary material for: EffectorFisher: association of disease phenotype with pangenomic protein-isoform profiles for improved prediction of fungal pathogenicity effectors
Source: Sci Rep. 2026 Mar 11;16:13077. doi: 10.1038/s41598-026-43646-x (PMC13100121; doi:10.1038/s41598-026-43646-x)
Supplement: Supplementary file 1 — Supplementary Material 1 [file 41598_2026_43646_MOESM1_ESM.pdf]

Supplementary Figures

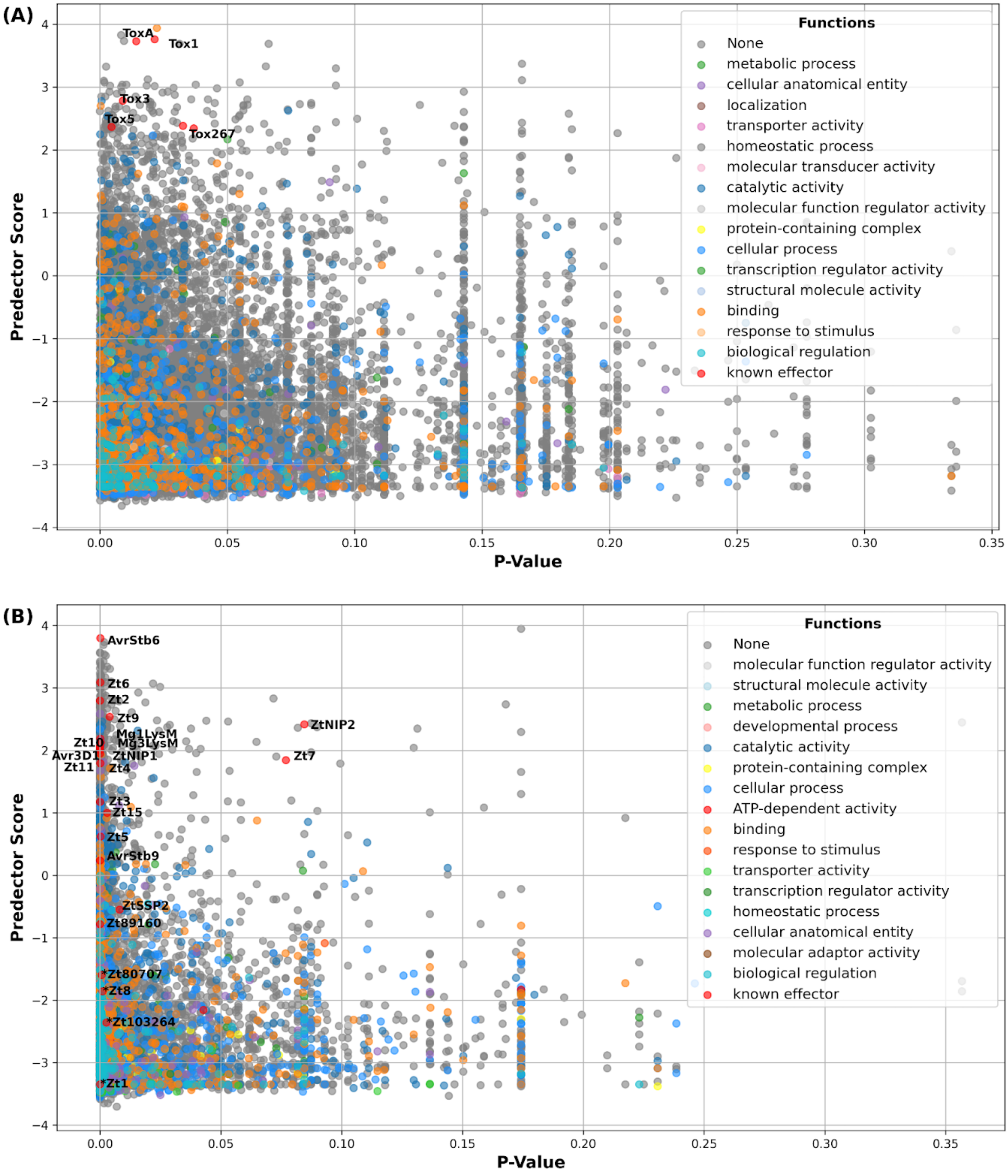

Supplementary Figure 1: Predicted whole-proteome of the necrotroph *Parastagonospora nodorum* (A) and the hemibiotroph *Zymoseptoria tritici* (B), indicating the distribution of EffectorFisher (phenotype-association) p-values (x-axis) relative to Predector scores indicating effector-like properties (y-axis). Broad functional annotation categories are color-coded (see legend) with confirmed effectors indicated by a red star.

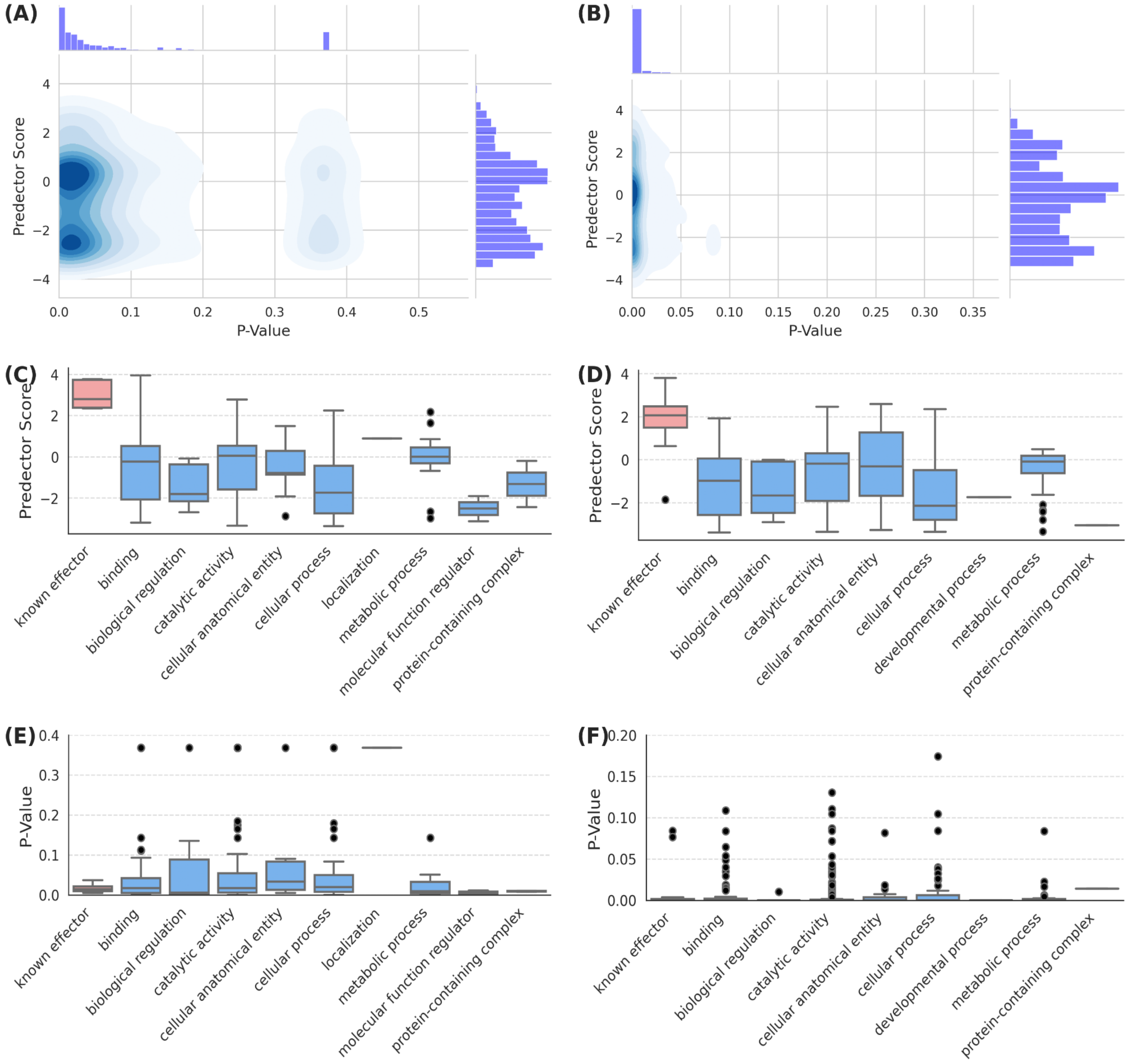

Supplementary Figure 2: Secretome distributions of EffectorFisher p-values (indicating disease phenotype association) and Predector scores (indicating effector-like properties), shown by density plots for A) the *Parastagonospora nodorum* pangenome versus Phenotype table B, and B) the *Zymoseptoria tritici* pangenome versus Phenotype table D; with box-whisker plots categorised by functional groups for *P. nodorum* Predector scores (C) and EffectorFisher p-values (E); and *Z. tritici* Predector scores (D) and EffectorFisher p-values (F).

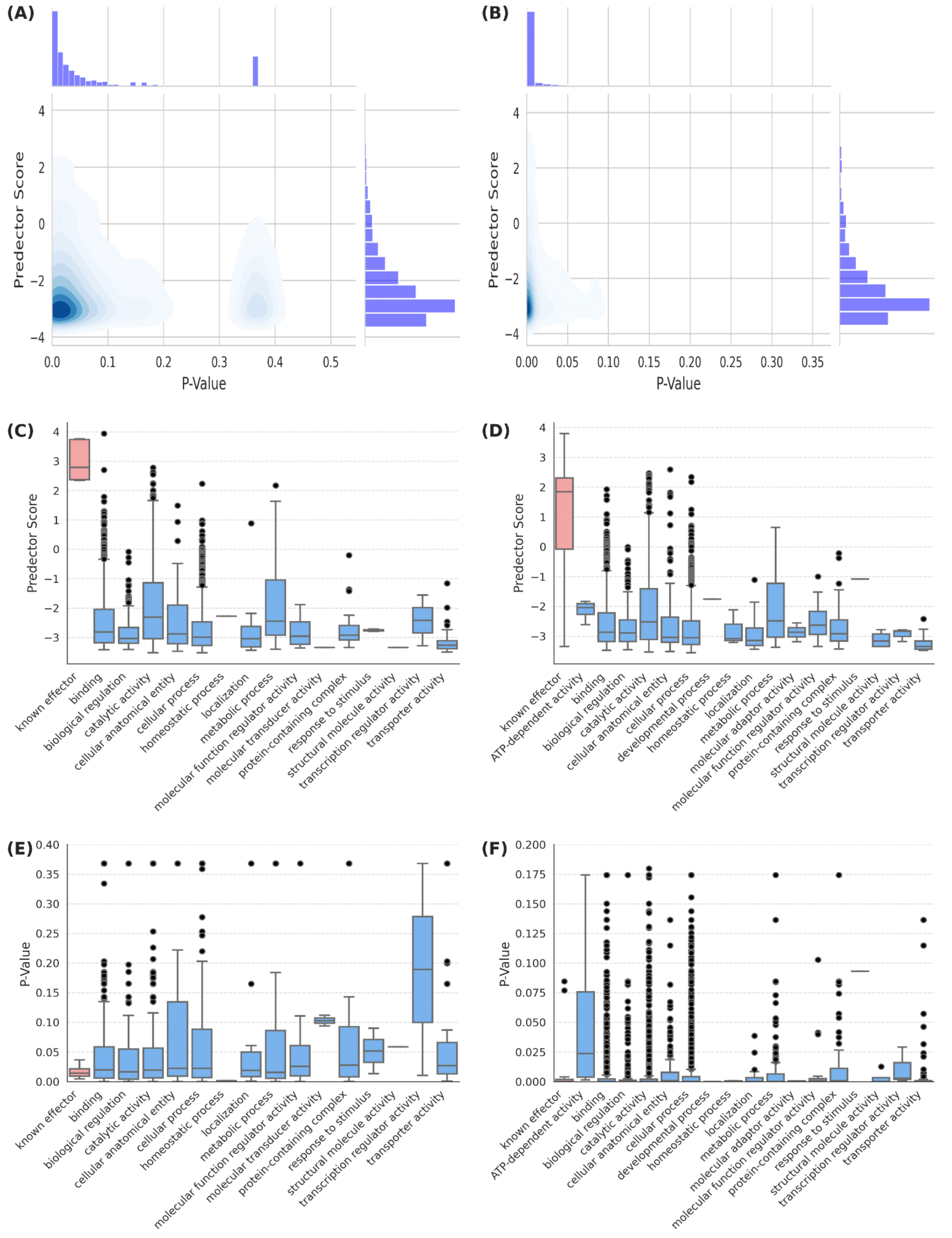

Supplementary Figure 3: Whole-proteome distributions of EffectorFisher p-values (indicating disease phenotype association) and Predector scores (indicating effector-like properties), shown by density plots for A) the *Parastagonospora nodorum* pangenome versus Phenotype table B, and B) the *Zymoseptoria tritici* pangenome versus Phenotype table D; with box-whisker plots categorised by functional groups for *P. nodorum* Predector scores (C) and EffectorFisher p-values (E); and *Z. tritici* Predector scores (D) and EffectorFisher p-values (F).

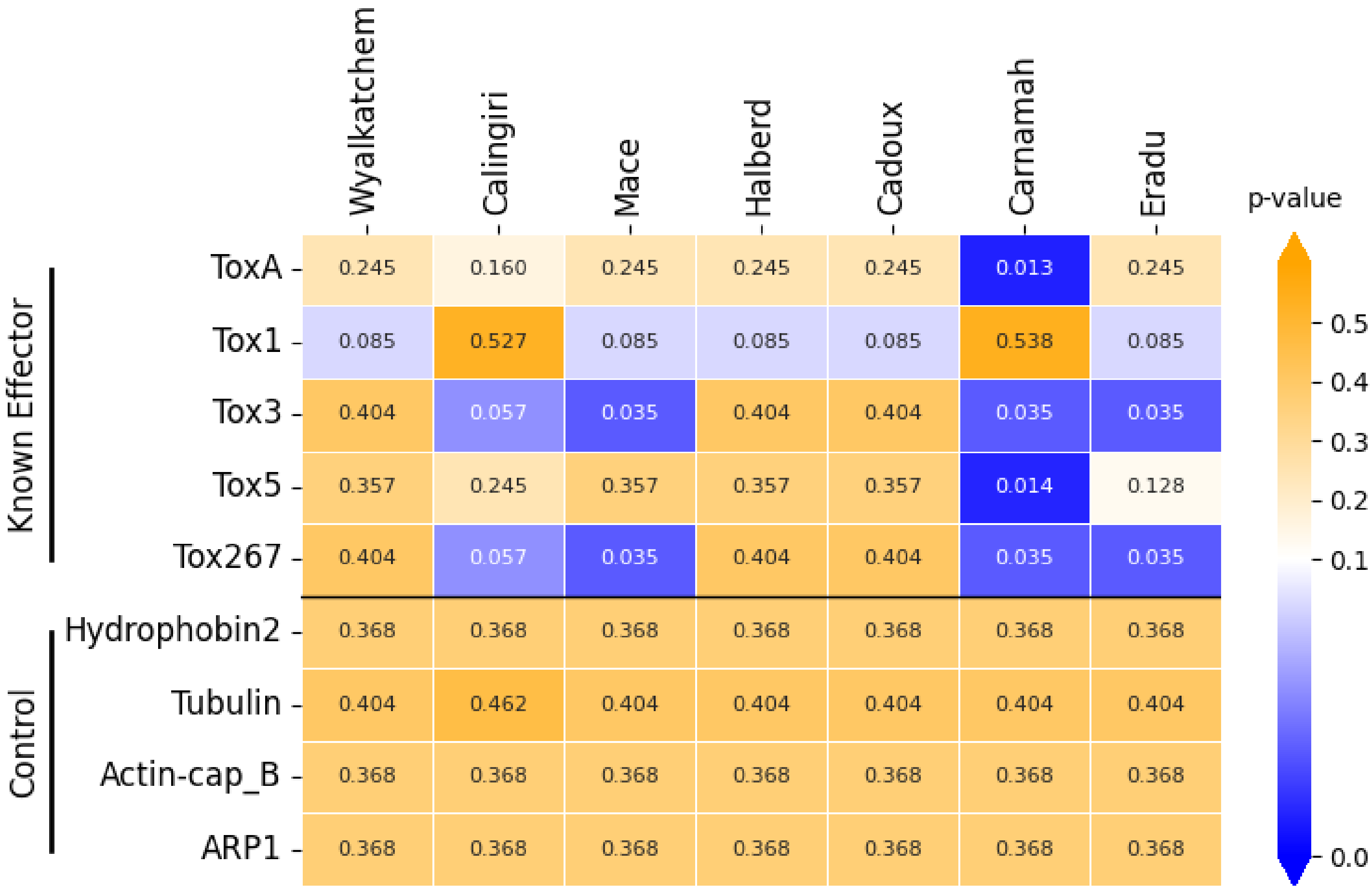

Supplementary Figure 4: Cultivar-specific association analysis p-values of *P. nodorum* known necrotrophic effectors vs non-effectors, summarised at the locus level, versus disease phenotype panel “phenotype-A”.

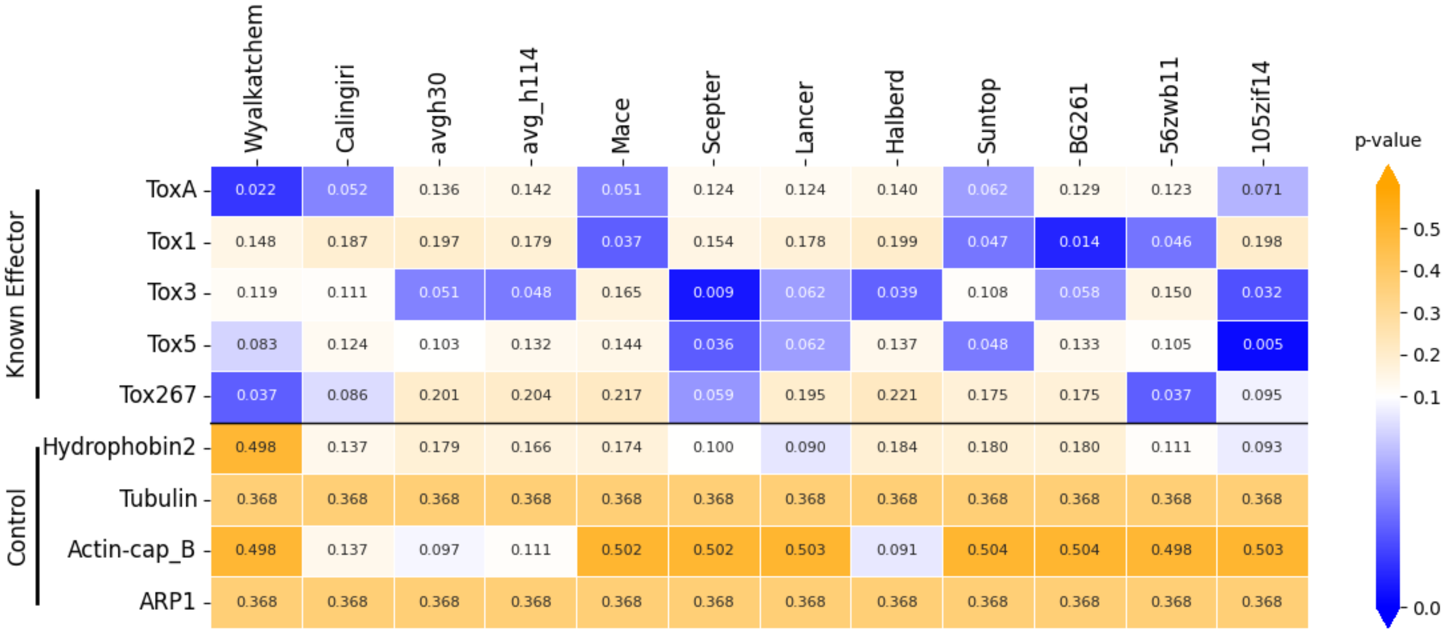

Supplementary Figure 5: Cultivar-specific association analysis p-values of *P. nodorum* known necrotrophic effectors vs non-effectors, summarised at the locus level, versus disease phenotype panel “phenotype-B”.

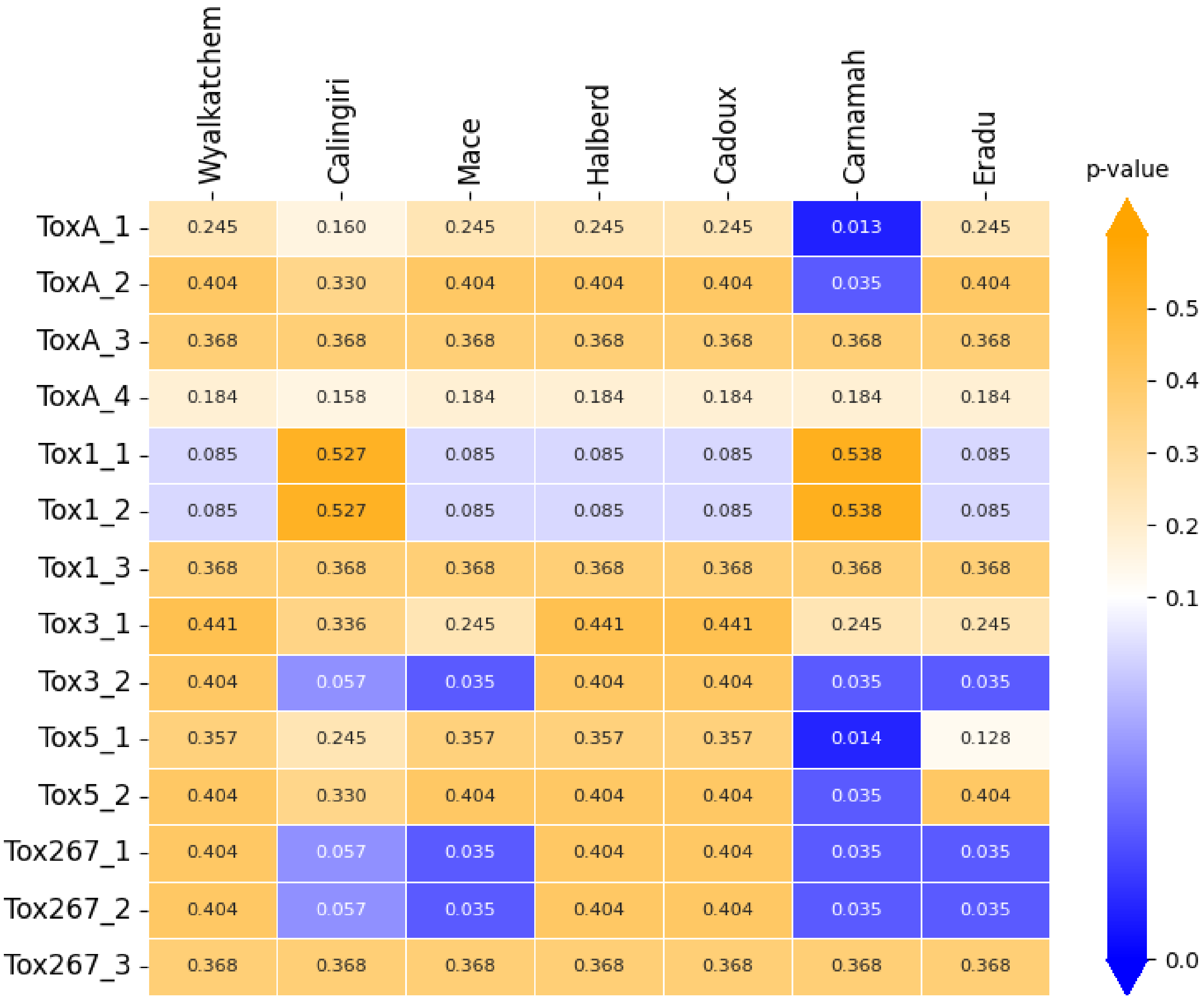

Supplementary Figure 6: Cultivar-specific association analysis p-values of individual protein-isoforms (unfiltered) of *P. nodorum* known necrotrophic effectors, versus disease phenotype panel “Phenotype-A”.

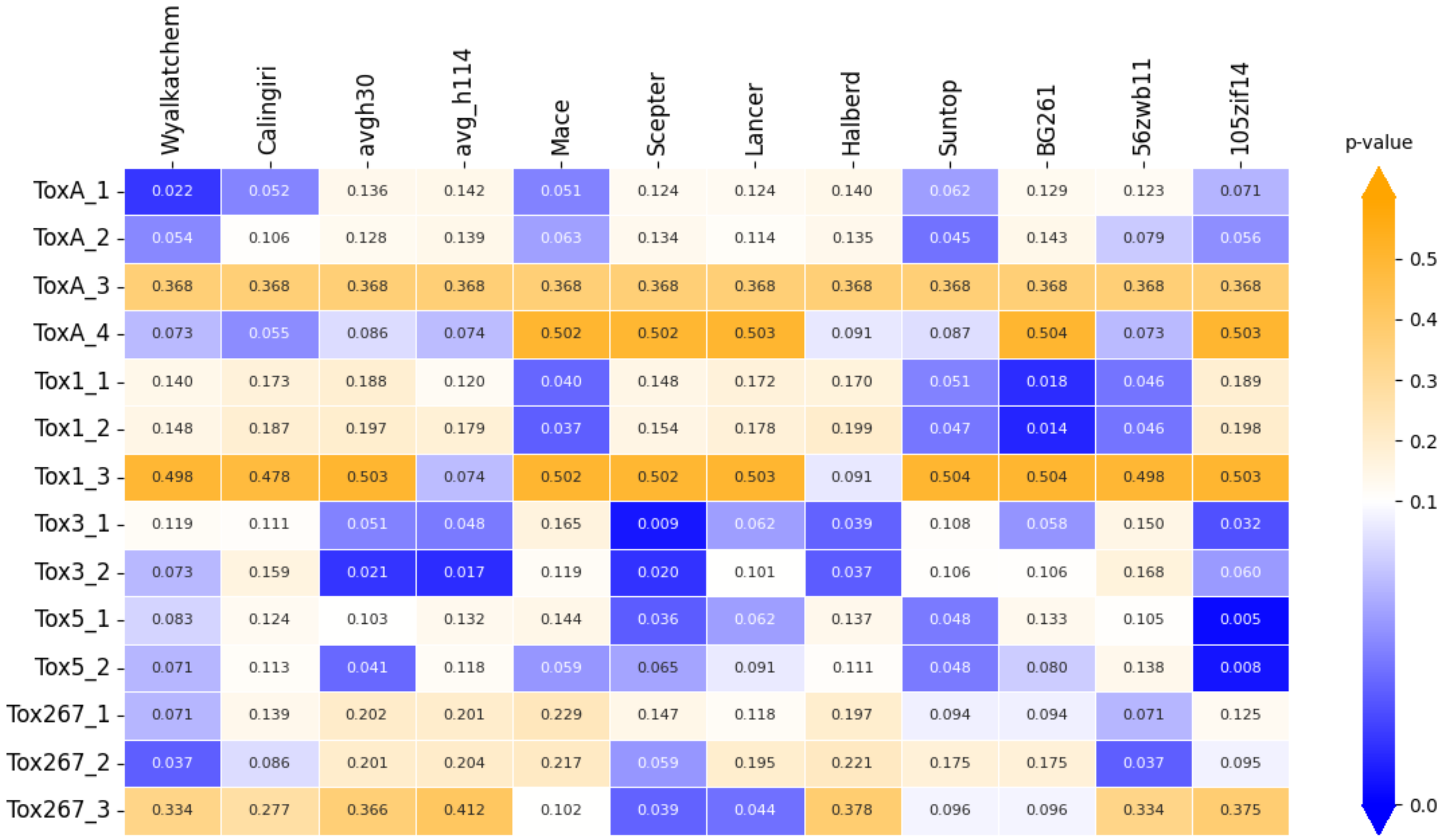

Supplementary Figure 7: Cultivar-specific association analysis p-values of individual protein-isoforms (unfiltered) of *P. nodorum* known necrotrophic effectors, versus disease phenotype panel “Phenotype-B”.

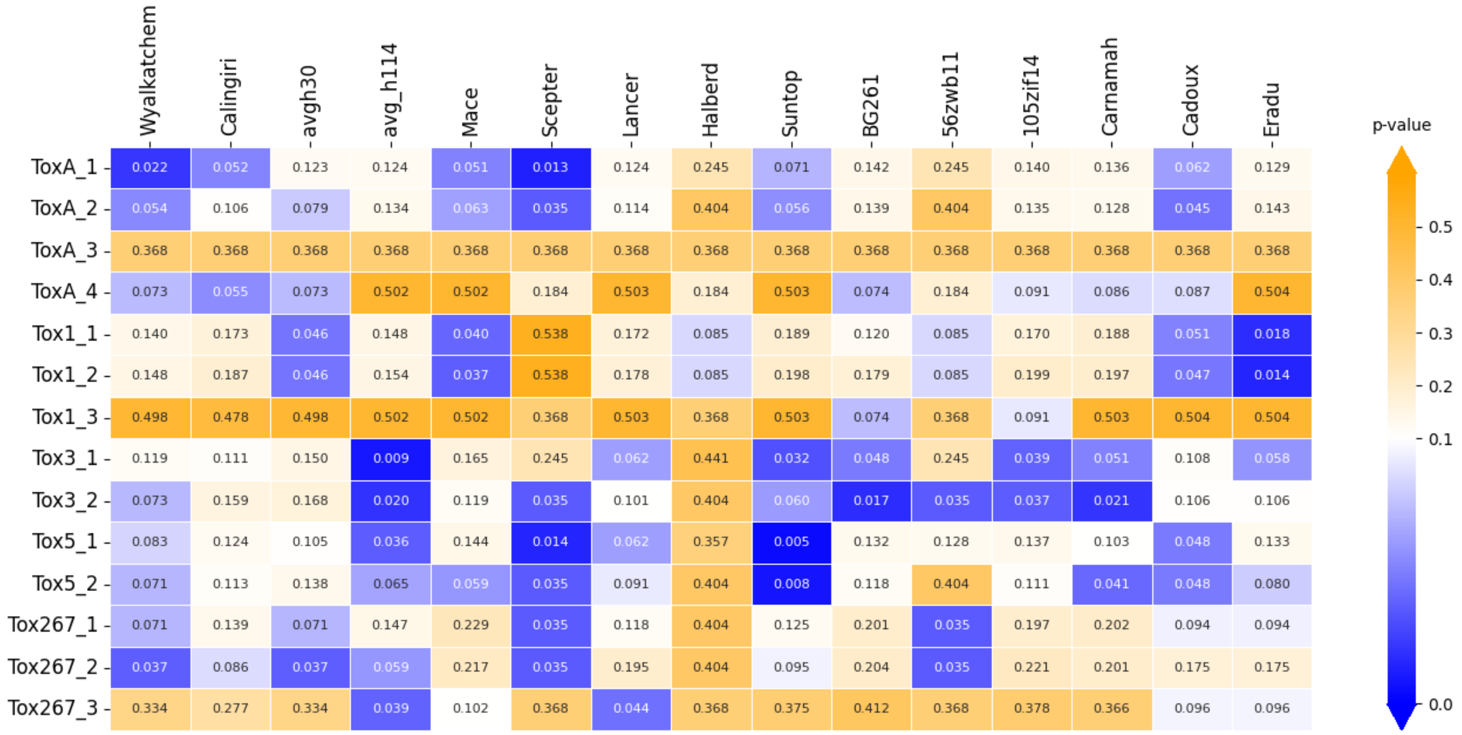

Supplementary Figure 8: Cultivar-specific association analysis p-values of individual protein-isoforms (unfiltered) of *P. nodorum* known necrotrophic effectors, versus disease phenotype panel “Phenotype-C”.

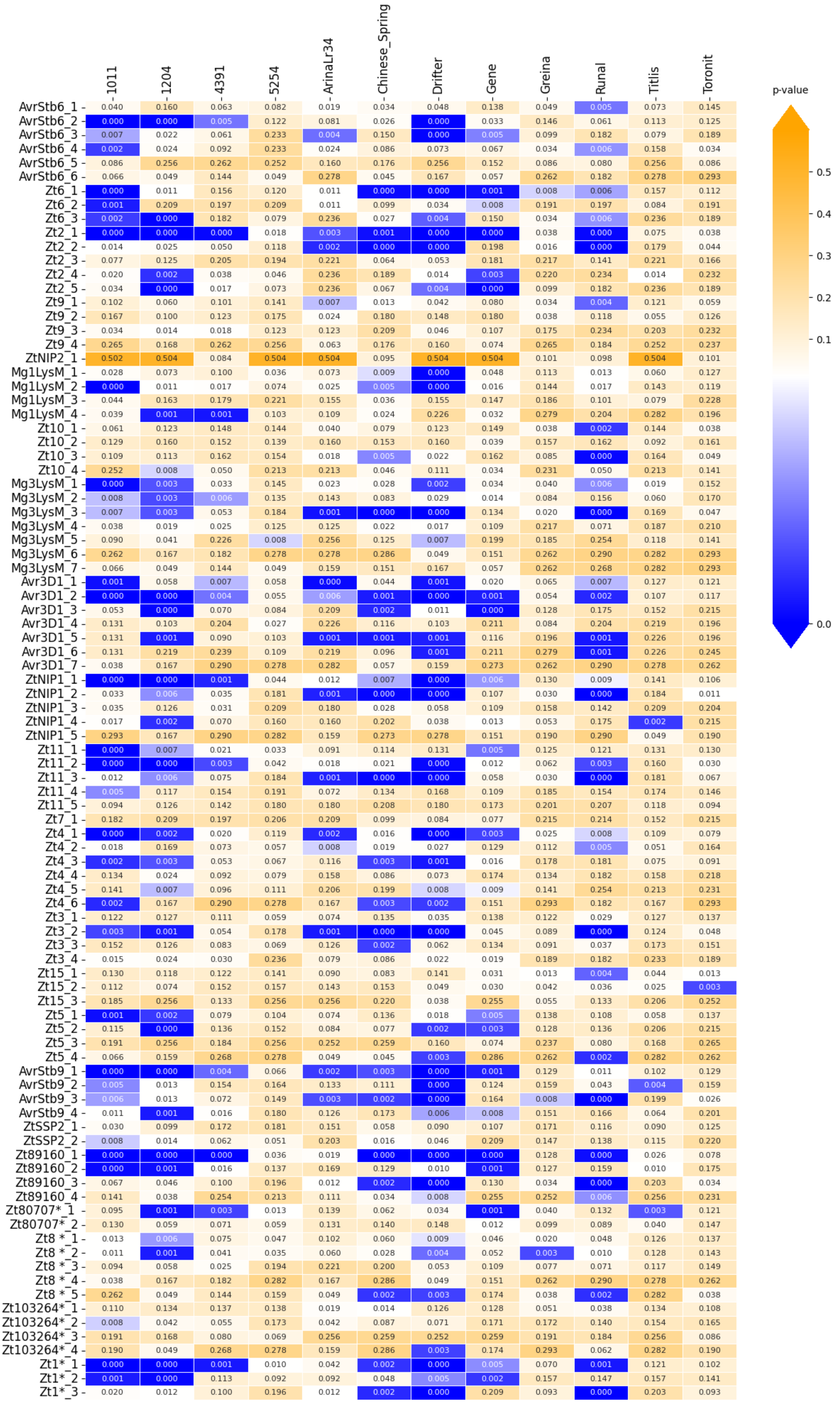

Supplementary Figure 9: Cultivar-specific association analysis p-values of individual protein-isoforms (unfiltered) of *Z. tritici* known necrotrophic effectors, versus disease phenotype panel “Phenotype-D”.

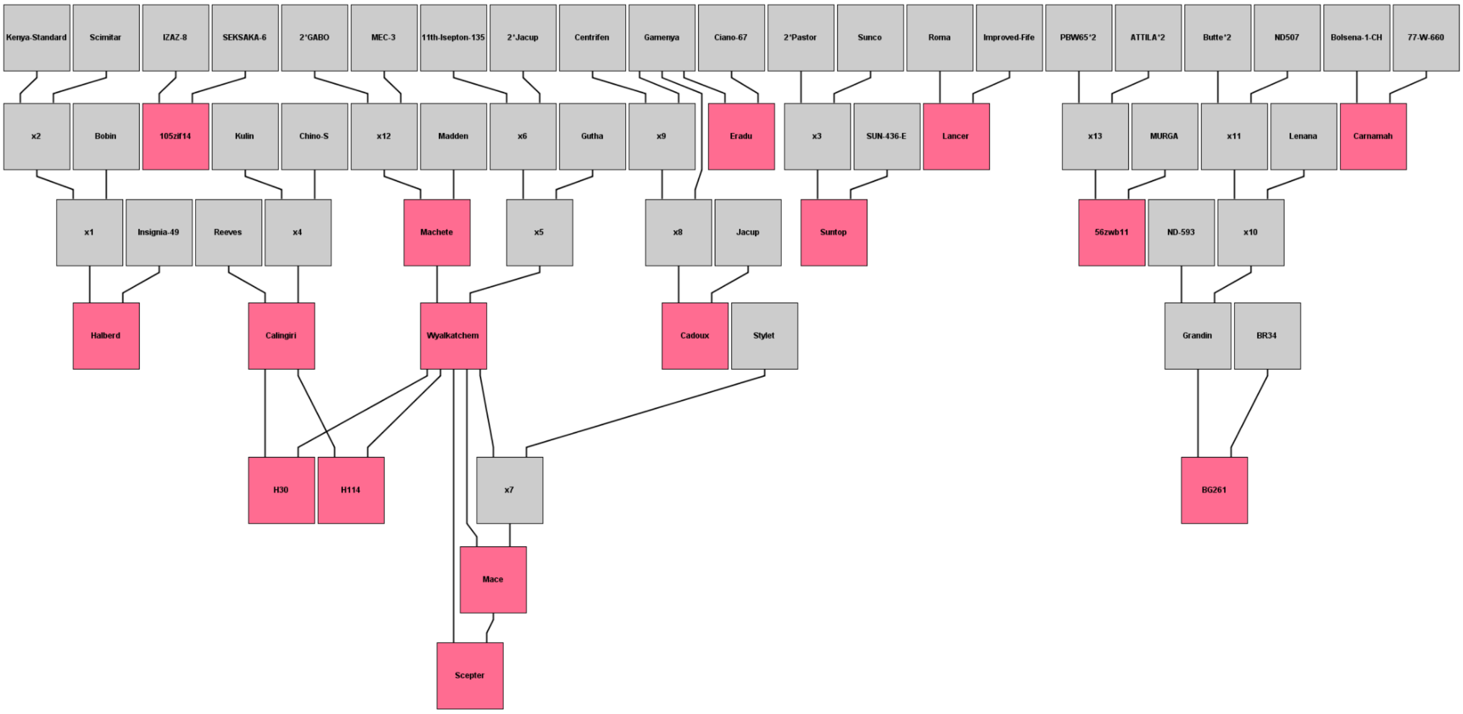

Supplementary Figure 10: Pedigree relationships between wheat cultivars used in *P. nodorum* the phenotype panels A-C (magenta).

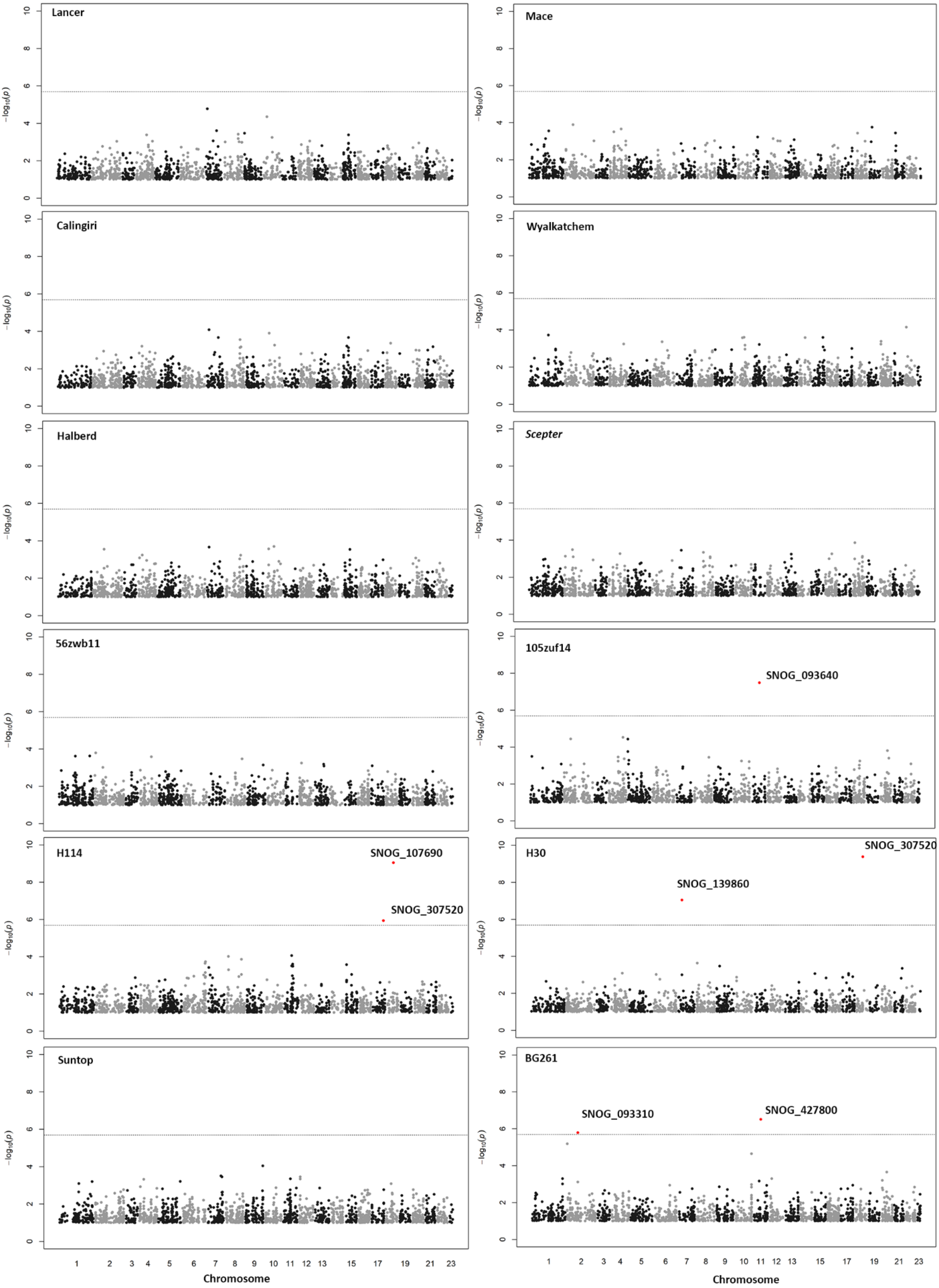

Supplementary Figure 11: Cultivar-specific GWAS Manhattan plots, showing disease phenotype-association p-values from multi-locus linear mixed models, for *P. nodorum* SNPs (LD-pruned) versus the "Phenotype-B" panel. The horizontal line indicates the Bonferroni threshold at a 5% significance level, with red dots representing SNPs with significant association and labels for its corresponding gene locus.

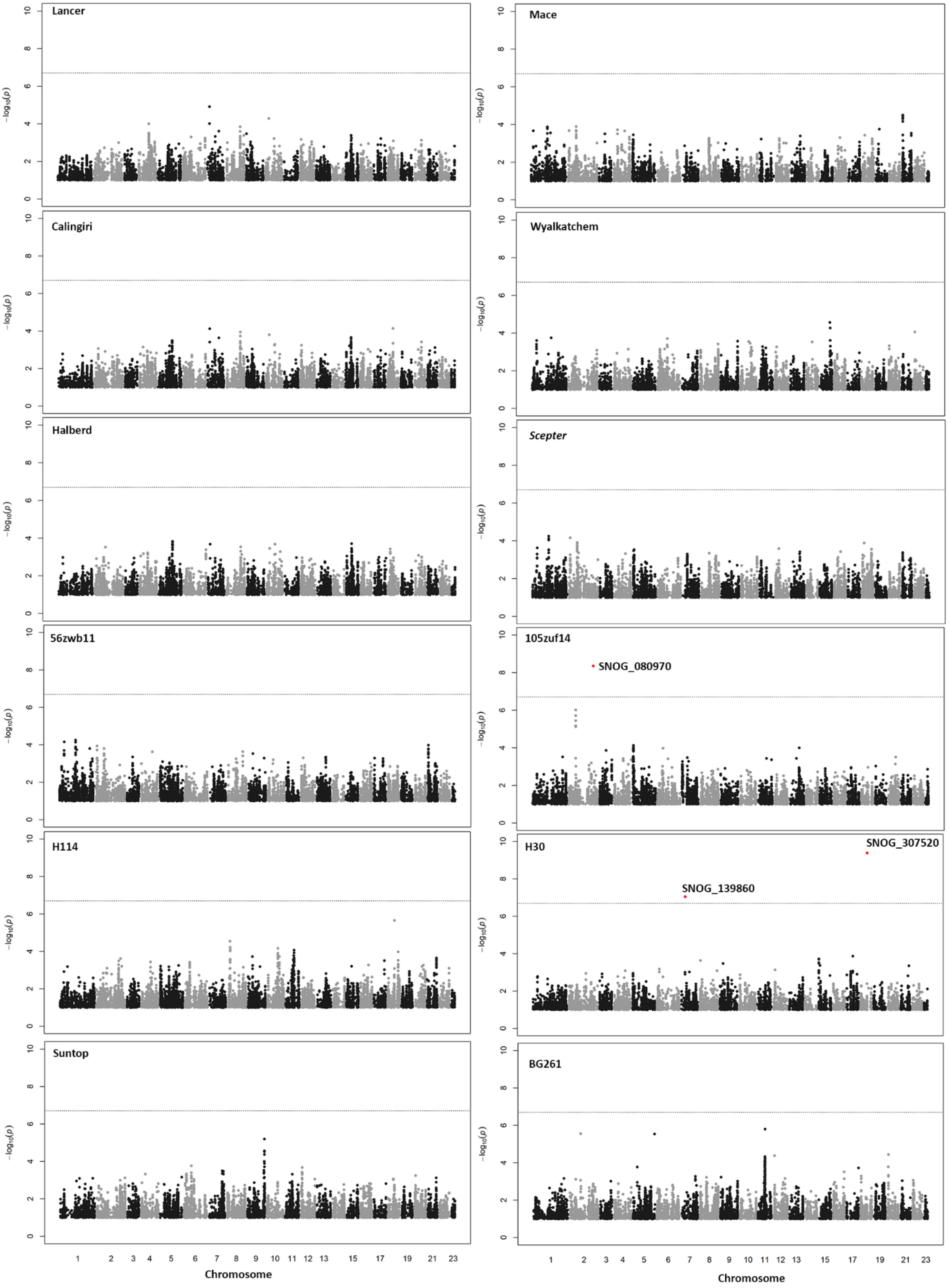

Supplementary Figure 12: Cultivar-specific GWAS Manhattan plots, showing disease phenotype-association p-values from multi-locus linear mixed models, for *P. nodorum* SNPs (without LD-filtering) versus the “Phenotype-B” panel. The horizontal line indicates the Bonferroni threshold at a 5% significance level, with red dots representing SNPs with significant association and labels for its corresponding gene locus.

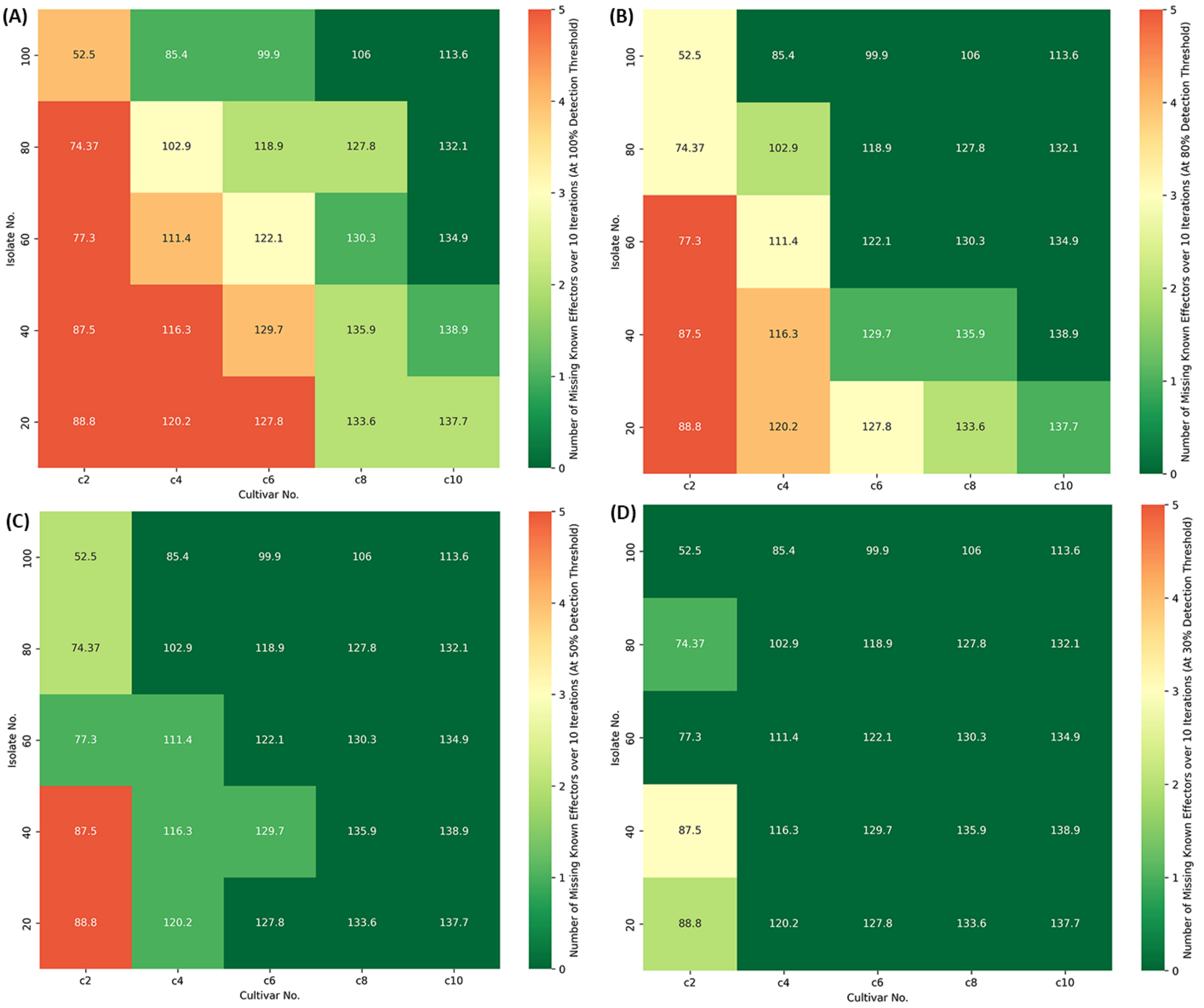

Supplementary Figure 13: The effects of different detection thresholds (missing = absence in 100%, 80% 50% or 30% of iterations), on the average of total CSEP predictions (cell values) and proportion of missing known effectors (all present =green, all missing=red) for different combinations of cultivars and isolates (for EffectorFisher p-value ≤ 0.1).

Supplementary Tables

Supplementary Table 1: Disease phenotyping panels used in this study
(see separately uploaded file)

Supplementary Table 2: Summary of GWAS analysis for P. nodorum versus phenotype panel “Phenotype-B”, showing *P. nodorum* ortholog groups that corresponded to significant SNPs.

Locus_id	SNPs-with/without-LD	Wheat Cultivar	EffectorFisher p-value	Predector Score	SN15_exp_up	Effector matches	pfam ids	pfam names
SNOO_093310	with LD filtering	BG261	0.141448953	-1.008	0	.	PF01680	Domain:SOR_SNZ
SNOO_307520	with and without LD filtering	H30, H114	0.000931608	-1.197	0	.	.	.
SNOO_139860	with and without LD filtering	H33	0.007091846	-2.203	0	.	PF00149	Domain:Metallophos
SNOO_093640	with LD filtering	105zuf14	8.23007E-05	-2.714	1	.	PF00144	Domain:Beta-lactamase
SNOO_107690	with LD filtering	H114	0.005115945	-2.729	0	.	PF14671,PF00782	Domain:DSPn,Domain:DSPc
SNOO_080970	without LD filtering	105zuf14	1.94095E-05	-2.948	0	.	PF06775	Family:Seipin
SNOO_427800	with LD filtering	BG261	0.032218172	.	0	.	.	.

Supplementary Table 3: Ranking of known *P. nodorum* necrotrophic effectors (based on predicted secretion, Predector score ≥2, protein length≤300, cysteine residues ≥2, and p-value≤0.1) using non-quantitative phenotype data.

Description	Effector-like Ranking (Secreted, Pred ≥2, Len≤300, Cys≥2)	Phenotype-association – Improved Ranking			Phenotype-association – Fold improvement		
		Simulated data (p≤0.1)	Simulated data (p≤0.05)	Phenotype- B (p≤0.05)	Simulated data (p≤0.1)	Simulated data (p≤0.05)	Phenotype- B (p≤0.05)
ToxA	3	2	2	3	1.50	1.5	1.00
Tox1	5	4	3	5	1.25	1.67	1.00
Tox3	40	25	21	25	1.60	1.90	1.60
Tox5	106	70	55	66	1.51	1.93	1.61
Tox267	110	74	-	69	1.49	.	1.59
Total candidates	433	126	101	117	3.41	4.28	3.70

Supplementary Table 4: Comparison of SnToxA protein-isoforms of this study with prior isoform and haplotype IDs from Tan et al. 2012 [50] and Aboukhaddor et al. 2023 [66].

ID (this study)	Position 99	104	114	121	135	137/8	163	166/7	Effector haplotype	Old name	Accession number
ToxA_1	E	V	N	N	E	L	I	S	ToxA3	H2	EF108458j
ToxA_2	E	I	N	N	E	L	I	S	ToxA10	H9	EF108459j
ToxA_3	E	I	D	R	E	V	I	T	.	.	.
ToxA_4	D	I	N	N	E	L	I	S	ToxA5	H4	EF108455j

Supplementary Table 5: Final predicted CSEP list for *P. nodorum,* versus 3 different phenotype panels (phenotype-A, phenotype-B, phenotype-C and non-quantitative phenotype data).
(see separately uploaded file)

Supplementary Table 6: Final predicted CSEP list for *Z. tritici,* phenotype panel “phenotype-D”.
(see separately uploaded file)

Supplementary Table 7: Isolate information and genome assembly metrics for *P. nodorum* (A) and *Z. tritici* (B) isolates used in this study.
(see separately uploaded file)

Supplementary Data

Supplementary Data 1: *P. nodorum* and *Z. tritici* isolates with genome sequence data and disease-phenotyping used in this study.
(see separately uploaded file)

Supplementary Data 2: Protein-isoform sequences of *P. nodorum isolates*.
(see separately uploaded file)

Supplementary Data 3: Protein-isoform sequences of *Z. tritici isolates*.
(see separately uploaded file)

Supplementary Data 4: Protein-isoform presence-absence profiles of *P. nodorum isolates*.
(see separately uploaded file)

Supplementary Data 5: Protein-isoform presence-absence profiles of *Z. tritici isolates*.
(see separately uploaded file)

Supplementary Data 6: Complete protein-isoform dataset with functional annotation, Predector effector prediction results, and EffectorFisher p-values, for *P. nodorum* versus the Phenotype-A dataset.
(see separately uploaded file)

Supplementary Data 7: Complete protein-isoform dataset with functional annotation, Predector effector prediction results, and EffectorFisher p-values, for *P. nodorum* versus the Phenotype-B dataset.
(see separately uploaded file)

Supplementary Data 8: Complete protein-isoform dataset with functional annotation, Predector effector prediction results, and EffectorFisher p-values, for *P. nodorum* versus the Phenotype-C dataset.
(see separately uploaded file)

Supplementary Data 9: Complete protein-isoform dataset with functional annotation, Predector effector prediction results, and EffectorFisher p-values, for *Z. tritici* versus the Phenotype-D dataset.
(see separately uploaded file)

Supplementary Data 10: Pedigree information for wheat cultivars included in Phenotype Datasets A-C.
(see separately uploaded file)
